# Metabolomic Study of Urine from Workers Exposed to Low Concentrations of Benzene by UHPLC-ESI-QToF-MS Reveals Potential Biomarkers Associated with Oxidative Stress and Genotoxicity

**DOI:** 10.3390/metabo12100978

**Published:** 2022-10-16

**Authors:** Michele P. R. Mendes, Maria José N. Paiva, Isabele C. Costa-Amaral, Leandro V. B. Carvalho, Victor O. Figueiredo, Eline S. Gonçalves, Ariane L. Larentis, Leiliane C. André

**Affiliations:** 1 Department of Clinical and Toxicological Analysis, Faculty of Pharmacy, Federal University of Minas Gerais (UFMG), Belo Horizonte 31270-901, MG, Brazil; 2 Center for the Study of Occupational Health and Human Ecology (CESTEH), Sergio Arouca National School of Public Health (ENSP), Oswaldo Cruz Foundation (Fiocruz), Rua Leopoldo Bulhões 1480, Manguinhos, Rio de Janeiro 21041-210, RJ, Brazil

**Keywords:** benzene, metabolomic untargeted, UHPLC-ESI-Q-TOF-MS, environmental toxicology, occupational toxicology

## Abstract

Benzene is a human carcinogen whose exposure to concentrations below 1 ppm (3.19 mg·m^−3^) is associated with myelotoxic effects. The determination of biomarkers such as *trans-trans* muconic acid (AttM) and *S*-phenylmercapturic acid (SPMA) show exposure without reflecting the toxic effects of benzene. For this reason, in this study, the urinary metabolome of individuals exposed to low concentrations of benzene was investigated, with the aim of understanding the biological response to exposure to this xenobiotic and identifying metabolites correlated with the toxic effects induced by it. Ultra-efficient liquid chromatography coupled to a quadrupole-time-of-flight mass spectrometer (UHPLC-ESI-Q-ToF-MS) was used to identify metabolites in the urine of environmentally (*n* = 28) and occupationally exposed (*n* = 32) to benzene (mean of 22.1 μg·m^−3^ and 31.8 μg·m^−3^, respectively). Non-targeted metabolomics analysis by PLS-DA revealed nine urinary metabolites discriminating between groups and statistically correlated with oxidative damage (MDA, thiol) and genetic material (chromosomal aberrations) induced by the hydrocarbon. The analysis of metabolic pathways revealed important alterations in lipid metabolism. These results point to the involvement of alterations in lipid metabolism in the mechanisms of cytotoxic and genotoxic action of benzene. Furthermore, this study proves the potential of metabolomics to provide relevant information to understand the biological response to exposure to xenobiotics and identify early effect biomarkers.

## 1. Introduction

Benzene is a ubiquitous pollutant, and human exposure to it occurs through occupational or environmental contamination. Benzene is used in the steel and oil industries, in addition to being released into the environment by emissions from motor vehicles (concentrations can reach up to 349 µg m^−3^ in high-traffic urban centers). In addition, cigarettes are another important anthropogenic source of environmental exposure [1]. It is a xenobiotic classified as a human carcinogen by the International Agency for Research on Cancer—IARC [2]. Exposure to benzene is associated with myelotoxic effects, which can cause qualitative and quantitative disturbances of blood cells such as leukopenia, thrombocytopenia, pancytopenia, aplastic anemia, and leukemia [3]. Studies have shown that benzene toxicity is greater at exposures associated with airborne concentrations below 0.1 ppm [4,5]. However, the mechanisms involved in its toxic action are still not fully elucidated.

Considering the potential risk to human health, biological monitoring is a useful exposure assessment tool to prevent adverse effects. Usually the biomarkers *trans, trans* muconic acid (A*tt*M) and *S*-phenylmercapturic acid (*S*-PMA) in urine are used for this purpose [6,7,8]. However, this strategy does not reflect the adverse health effects resulting from exposure to low concentrations of this toxic agent. Therefore, an exploratory and comprehensive evaluation of the organism-benzene interaction is necessary; and metabolomics present itself as a useful alternative capable of achieving this goal.

Biomonitoring is used to indicate the occurrence of an internal dose of the chemical and its distribution in the body; which can be useful in measuring health risks. Currently, the assessment of risks arising from chronic exposure to low concentrations of toxic substances or complex mixtures represents a major challenge for modern toxicology. In many cases, the quantitative determination of exposure and effect biomarkers has been shown to be insufficient to fully characterize the harmful effects resulting from this exposure. Added to this, the inability of biomarkers to early indicate the harmful effects induced by the toxic agent [9].

In this sense, the application of “omics” sciences for toxicological evaluation has been proposed and in this context, metabolomics has stood out among the most promising [10,11]. Metabolomics consists of the comprehensive assessment of the metabolic profile of biological compartments and represents an important step toward the discovery of new biomarkers associated with environmental or occupational exposure. However, the limited number of available metabolomic biomarkers does not reflect the extent of research in this field. Among the challenges of metabolomics, we can mention the analytical process, which is critical, in addition to the difficulties in reproducing the data necessary for the replication of studies, or even its continuity after the discovery of biomarkers [12].

Disturbances in metabolic profiles are detected earlier when compared to alterations in genes or proteins [13,14]. Thus, in the field of toxicology, metabolomics represent a holistic understanding of the interaction between organism and xenobiotic, and allows the integration of information about the internal dose of the chemical and the biological response triggered, essential data to assess potential risks [15,16]. Therefore, the metabolomics approach is a powerful tool to deepen our understanding of the biochemical interactions involved in disease development from chemical exposures to the discovery of new biomarkers.

The comprehensive coverage of the chemical diversity of substances and their different concentrations in the human body is a challenge, and for this the concomitant use of different analytical techniques is necessary. Nuclear magnetic resonance—NMR [17,18] and MS-mass spectrometry [19] account for most applications. However, MS offers greater sensitivity and specificity when combined with separation techniques such as liquid or gas chromatography. Liquid chromatography is recognized as the most used technique currently in non-target metabolomics [20,21].

Untargeted metabolomics has been successfully applied in studies of environmental and occupational toxicology, providing relevant information on the mechanisms of toxic action and even identification of biomarkers [22,23,24,25,26,27,28]. However, there are few studies on the elucidation of the metabolic profile in individuals exposed to benzene.

In this study, an exploratory experimental analysis was performed in order to identify whether there are differences in the urinary metabolic profile of individuals occupationally and environmentally exposed to low concentrations of benzene. For the study of the metabolome, ultra-performance liquid chromatography coupled to a quadrupole-time-of-flight electrospray ionization mass spectrometer (UHPLC-ESI-Q-TOF-MS) was used. Multivariate statistical analysis was used to analyze the results and understand the biological response to exposure and, thus, encourage future studies in the search for new biomarkers; more specific and capable of predicting the adverse effects caused by exposure to this chemical contaminant. These new biomarkers can be used in the context of environmental and occupational health, with a preventive purpose, in order to prevent exposed individuals from becoming ill.

## 2. Materials and Methods

### 2.1. Population

The study population consisted of 60 workers of both sexes, aged between 18 and 65 years, with a history of exposure to low concentrations of benzene. These workers were part of the population described in the study by Costa-Amaral et al., (2019) [29]. The workers were divided into two groups, according to their exposure to benzene—environmental exposure and occupational exposure. The environmental group was composed of 28 workers who worked as security guards (environmental group) of a research institution located in the north of the city of Rio de Janeiro-Brazil. The occupational group included 32 gas station workers located in the west of the same city.

### 2.2. Ethical Aspects of Research

Urine samples from the 60 subjects were provided, in accordance with the “Ethical Principles for Medical Research Involving Human Subjects” of the World Medical Association, by the Center for Studies in Occupational Health and Human Ecology of the Oswaldo Cruz Foundation (CESTEH/FIOCRUZ), in Rio de Janeiro. of January Brazil. These samples were part of the FIOCRUZ study on the assessment of occupational exposure to benzene. The study was submitted and approved by the ethics and research committees of the Federal University of Minas Gerais—UFMG (CAAE: 57396116.1.0000.5149, opinion number: 1.691.348) and of FIOCRUZ-RJ (CAAE: 17438013.5.0000.5240, opinion number: 2.974.839).

### 2.3. Biochemical Biomarkers, Exposure, Oxidative Stress and Genotoxicity

Blood samples were collected to determine hematological parameters (complete blood count + platelets) and biochemical parameters (AST, ALT, total bilirubin and fractions, creatinine, GGT); which were analyzed by the Ambulatory of the National School of Public Health—ENSP/Fiocruz-RJ. The biomarkers of oxidative stress effect (enzyme activity: catalase—CAT; glutathione S transferase—GST; superoxide dismutase—SOD; thiol and malondialdehyde -MDA Group) and genotoxicity (micronucleus, chromosomal aberrations) were performed at the Toxicology Laboratory from the Center for the Study of Workers’ Health and Human Ecology—CESTEH/FIOCRUZ (COSTA-AMARAL, 2019) [29]. The comet assay (EC), another biomarker of genotoxicity; was carried out in the laboratory of Professor Dr. Andrew Richard Collins linked to the Department of Nutrition at the University of Oslo-Norway (COSTA-AMARAL, 2019) [29]. Urine samples were collected for quantification of benzene exposure biomarkers SPMA and AttM, and global metabolomics. AttM was also quantified in the Toxicology Laboratory of CESTEH/FIOCRUZ; while the SPMA and metabolomics analyzes were performed at the Laboratory of Toxicological Analysis (LATO) of the Faculty of Pharmacy at UFMG.

### 2.4. Standards and Reagents

The chromatographic grade solvents acetonitrile, methanol, formic acid, 2-propanol, and sodium formate were obtained from Sigma-Aldrich (San Luis, MO, USA)^®^.

### 2.5. Sample Preparation

Urine samples were frozen at −80 °C to promote metabolic extinction. To perform the analyses, the urine samples were thawed and vortexed for 30 s. Then, 100 µL were diluted in 125 µL of methanol in Eppendorf microtubes and again vortexed for 30 s. The samples were then subjected to centrifugation at 2817× *g* at 4 °C for 5 min for protein precipitation. Soon after, 200 µL of the supernatant were transferred to properly labeled flasks and diluted in 300 µL of an aqueous solution containing 0.1% methanoic acid. Quality control samples (QCs) were prepared by mixing 20 µL of each urine sample. Sample blanks were also prepared containing all reagents used in the preparation, with the exception of urine.

### 2.6. Analytical System—UHPLC-ESI-QTOF/MS

For data acquisition, after preparation, urine samples were analyzed by ultra-performance chromatography (NCS-3500RS-Thermo Scientific^®^ model) coupled to a mass spectrometer (Bruker^®^ Q-ToF model) and Bruker^®^ HMDB library Metabolite 2.0. A C18 column (100 mm × 2.1 mm 1.8 µm particle) was employed in the analysis, the temperature was maintained at 40 °C with gradient elution and low flow, as shown in Table 1. The mobile phase consisted of mixing the solutions (A): water with 0.1% formic acid and (B): acetonitrile with 0.1% formic acid. The injection volume was 15 µL. Quality control (QC) samples were interleaved every five urine samples to assess instrument stability.

The mass spectrometer was operated in positive mode, with electrospray ionization (IE), using collision energies of 20 and 50 eV (each 50% of the time). The ionization source was adjusted to 4500 V in positive mode with a potential plate end of −500 V. The source conditions was adjusted to: nebulizer gas (N_2_) at 5.0 bar; dry gas (N_2_) at 9.0 L/min; dry temperature at 200 °C; capillary voltage at 4.5 KV. Before analysis, sodium formate solution was used for calibration. Lock mass calibration was performed with 1 mg/mL solution of hexakis (2,2-difluoroethoxy) phosphazene in 2-propanol, generating reference ions for positive ion mode ([M + H]^+^ = m/z 622).

Data were obtained in a range of m/z 50 to 1200 with an acquisition rate of 4 Hz. Five most intense ions were selected for automatic fragmentation (AutoMS/MS). Data were acquired by the Hystar Application^®^ software version 3.2 and OtofControl^®^ (Bruker Daltonics Corporation, Bremen, Germany) and processed using DataAnalysis^®^ 4.4 and Metaboscape^®^ 5.0 softwares (Bruker Daltonics Corporation, Bremen, Germany).

All samples were analyzed randomly in order to avoid uncertainties and artifacts related to the injection order and to prevent the effect of gradual changes in instrument sensitivity over entire batches.

### 2.7. Detection and Identification of Non-Target Metabolites

The results files obtained from anlytical system were converted to the mzXML format using the Proteowizard^®^ software (http://proteowizard.sourceforge.net/download.html (accessed on 27 January 2020)). The R^®^ statistical software version 3.6.2 and the XCMS Bioconductor^®^ package (Mahieu, Genenbacher, & Patti, 2016; version 3.10 http://bioconductor.org/packages/release/bioc/html/xcms.html (accessed on 27 January 2020)) [30,31] were used for the pre-treatment of the data and creating the data matrix. The results were analyzed individually using the algorithm centWave in XCMS^®^. The data were submitted to logarithmic transformation and Pareto scheduling. The peak intensities of each sample were normalized by the corresponding creatinine concentration; using MetaboAnalyst^®^ version 5.0 (https://www.metaboanalyst.ca/ (accessed on 27 January 2020)) [32].

Multivariate statistical analysis using the principal component analysis (PCA) and discriminant analysis by partial least squares (PLS-DA) tests was used to identify the metabolic differences in the urine of both groups. Cross-validation were used to validate the PLS-DA model. All of these analyzes, as well as the identification of the metabolites as potential biomarkers, were also performed in MetaboAnalyst^®^ 5.0, using the following criteria: variable importance plot (VIP) scores of the variables > 2; *p*-values between analyzed groups < 0.05, FDR < 0.01 and metabolites significantly correlated with oxidative stress and genotoxicity biomarkers (*p* < 0.05).

Compounds identification was performed with a narrow tolerance of 3.0 mDa for mass accuracy, mSigma of 40 for isotope standard and MS/MS score of 700, using Bruker Metabobase Personal Library 3.0^®^ and Bruker HMDB Metabolite Library 2.0^®^ (MSI level 2 [33]). For unidentified metabolites, molecular formulas were generated using Compound Crawler (Metaboscape^®^ 5.0) with close tolerance of 3 mDa and mSigma of 30; considering CHNOPS as elements of composition. For these, the putative identification in public databases such as—Human Metabolome Database (HMDB^®^), Metlin^®^, and LIPIDMAPS^®^ was performed by searching the m/zs, based on the accurate mass and mass spectrometric fragmentation patterns, and maximum mass error of 5 ppm.

### 2.8. Statistical Analysis

The Shapiro–Wilk test was used to assess whether the quantitative variables were normally distributed. To compare the normally distributed variables, the T test was used; for the others, the Mann–Whitney test was used. To compare the qualitative variables, the Chi-Square and Fisher’s Exact tests were used. Differences in urinary metabolite concentration between groups were investigated using the Mann–Whitney test. Multiple linear regression was used to explain the variability of effect biomarkers as a function of urinary metabolites. The linear regression model used was:

$$Y_{i}=\beta_{0}+\overset{p}{\underset{k=1}{\sum }}\beta_{k}\;X_{ik}+\epsilon_{i}{}^{}$$

where

- $Y_{i}$ is the value of the oxidative stress biomarker of the ith individual;
- $X_{ik}$ is the kth metabolite of the ith individual;
- $\beta_{ik}$ is the coefficient of the kth metabolite;
- $\epsilon_{i}$ is a random error, which follows normal distribution with mean 0 and standard deviation σ.

Through the regression study, we sought to identify the most important urinary metabolites to explain the variance of the oxidative stress biomarker. The stepwise backward elimination [34] method, using the Akaike Information Criterion [35], or AIC, as a criterion was used to identify the combination of metabolites that minimized the AIC and led to a more adjusted model, capable of explaining the variance of the effect biomarkers.

Finally, the correlation between metabolites and exposure biomarkers (SPMA and AttM, oxidative stress markers and chromosomal aberration rates) was verified using Spearman’s Correlation and illustrated by scatter diagrams. The software used in the analyzes was R^®^ (version 4.0.2) and all statistical tests were performed assuming a significance level of 5%.

## 3. Results

### 3.1. Sociodemographic Characteristics and Results of Biological Monitoring

The descriptive analysis of sociodemographic characteristics and the results of the comparison tests between the groups of workers are shown in Table S1. The occupationally exposed population consisted of 72.5% gas station attendants; 13.7% employees with executive assignments; 5.9% general service assistants; 5.9% lubrication technician; and 2.0% convenience store clerk. The mean concentration of benzene in the atmospheric air of the evaluated stations was 31.8 μg·m^−3^. On the other hand, the population environmentally exposed to benzene was composed of security guards (96.6%) and doormen (3.4%), who were exposed to an average concentration of 22.1 μg·m^−3^ of benzene in the air atmosphere. All participants from both groups had been in this occupation for more than 3 months.

Working time in the current occupation varied greatly between groups, as shown in Table S2. In the environmental group, workers’ time in their current occupation ranged from 10 to 348 months, with a mean of 152.7 and a standard deviation of 99.2. In the occupational group, the working time ranged from 12 to 408, with a mean of 117.8 and a standard deviation of 122.8.

The complete description of the results of the descriptive analysis of the sociodemographic characteristics, hematological, biochemical parameters, oxidative stress, and genotoxicity of the workers participating in this study were previously described by Costa-Amaral et al., (2019) [29], and a summary is presented in Table S3. Oxidative stress biomarkers THIOL, MDA, and chromosomal abnormalities differed significantly between groups. Thus, oxidative damage and chromosomal aberrations were the main toxic effects induced by exposure to benzene in the population studied. For this reason, the correlation of potential metabolomic biomarkers in urine with such toxic effects was investigated in the present study.

### 3.2. Identification of the Urinary Metabolic Profile of Individuals Occupationally and Environmentally Exposed to Benzene

**Figure 1:**
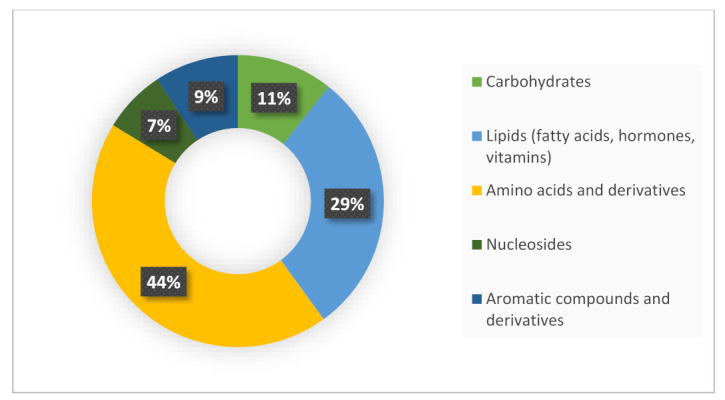
Chemical categories of metabolites identified in the urine of occupational and environmental workers exposed to benzene, investigated in this study.

A total of 2361 molecular characteristics were detected, of which 1522 showed significant differences (*p* < 0.05) between occupational and environmentally exposed groups. Of these, 1267 compounds were identified; which belong to different chemical categories, as shown in Figure 1. There was a predominance of amino acids and derivatives (44%) and lipids (29%).

**Figure 2:**
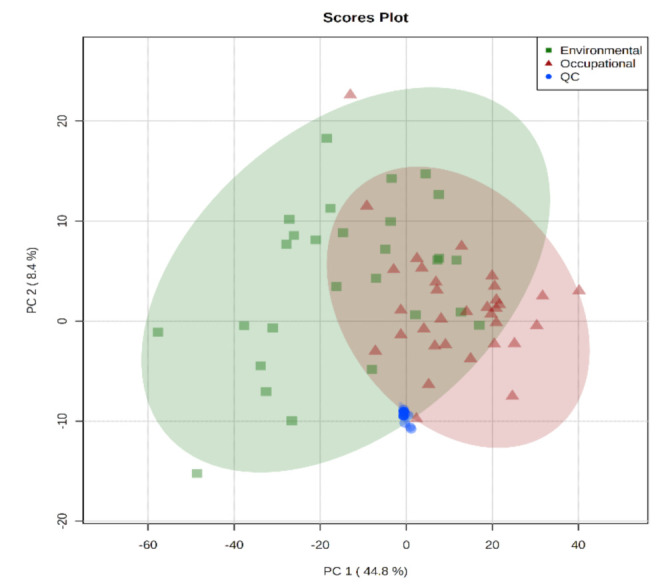
Principal component analysis-PCA (PC 1 × PC 2) of urine samples from workers exposed and occupationally to benzene, with provision for environmental quality control samples (QCs).

The analytical results were analyzed by unsupervised pattern recognition methods—PCA (principal component analysis) and supervised—PLS-DA (partial least squares discriminative analysis). PCA analysis was used to assess the quality of data acquisition, and the strong clustering of QC samples, observed in the center of the graph (Figure 2), indicated analytical reproducibility and reliability of the results. The PCA model constructed also revealed a tenuous separation between the groups of workers with low environmental and occupational exposure to benzene.

**Figure 3:**
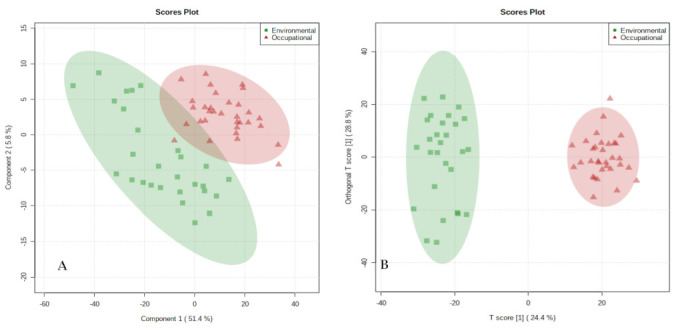
Partial least squares discriminant analysis (PLS−DA) and partial orthogonal discriminant analysis (OPLS−DA) of urine samples from workers exposed to environmental and occupational benzene, analyzed by UHPLC−ESI−Q−ToF−MS. Note: Red triangles represent samples from the occupational group and the green squares represent samples from the environmental group. (**A**): PLS−DA model, validation parameters: R^2^ = 0.98, Q^2^ = 0.87; (**B**): OPLS−DA model.

The supervised pattern recognition methods—discriminant partial least squares analysis (PLS-DA) and discriminant partial least squares analysis (OPLS-DA); were used to identify the metabolites capable of distinguishing the groups of workers investigated in this study. The PLS-DA model constructed is shown in Figure 3. The PLS-DA revealed a tendency to cluster samples from the same group; especially in the occupational group. This indicates that there are differences in the urinary metabolic profile between the groups of workers; and that the metabolic disorder associated with exposure differs between them. The constructed PLS-DA and OPLS-DA models were validated through the cross-validation test and the permutation test, respectively; and both presented satisfactory coefficients.

Based on the criteria for identifying the discriminating metabolites described in item 2.5, thirty-eight molecular characteristics that contributed most to the discrimination between groups were selected. Table 2 shows the identity of the metabolites, as well as their m/z ratio and chemical classification. The intensities of metabolites between groups were compared by the Mann–Whitney test, and all showed a statistically significant difference (*p* < 0.05). The VIP value, obtained in the PLS-DA model, indicates the importance of each metabolite in the discrimination between groups.

All metabolites showed higher concentration (fold change) in the urine of workers with environmental exposure to benzene, with the exception of the compound phenylalanylhydroxyproline, which showed higher concentration in the urine of gas station workers.

The correlation between potential metabolomic biomarkers and *S*-phenilmercapturic Acid (SPMA) and *trans-trans*-muconic acid (*t,t*-MA) exposure indicators was investigated by Spearman’s Correlation. No significant correlation was observed (*p*-value > 0.05) between metabolites and these biomarkers (Table S4).

### 3.3. Urinary Metabolites Associated with Oxidative Stress

The oxidative stress biomarkers SOD, CAT, and GST showed no significant difference between groups. To investigate the relationship of these discriminating metabolites derived from metabolomic analysis with the early toxic effects associated with low exposure to benzene identified in this study—thiol and MDA—linear regression was used. Thus, linear regression models were developed, using the stepwise backward elimination method and the Akaike information criterion (AIC), to explain thiol and MDA.

**Figure 4:**
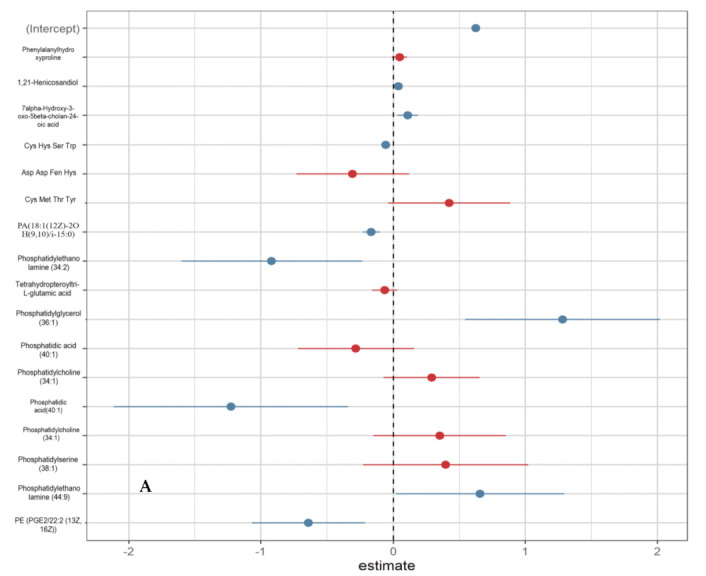

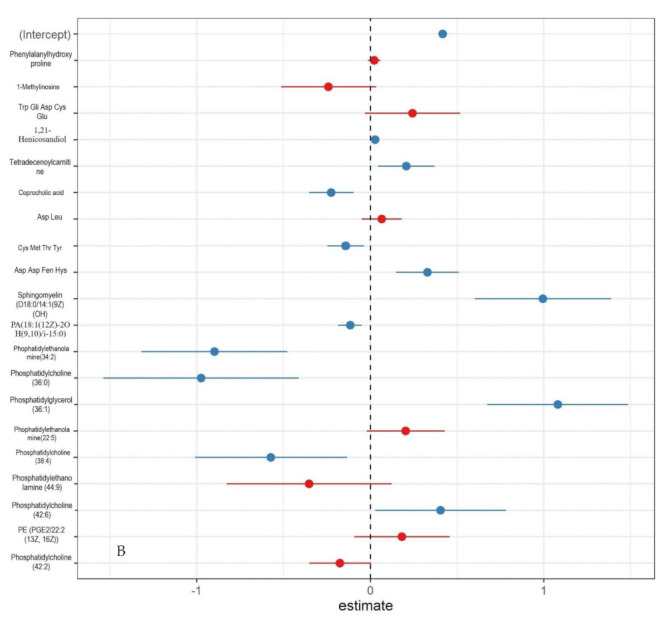
Logistic linear regression results with estimates of $\beta_{0}$ e of $\beta k_{}$ of discriminating metabolites for MDA and thiol biomarkers. Note: (**A**): MDA; (**B**): THIOL. Note: Coefficients calculated using linear regression, with a 95% confidence interval (CI). In blue, significant urinary metabolites; in red, non-significant urinary metabolites.

In Figure 4 it is possible to see the metabolites of the best linear regression model to explain MDA and thiol, and their respective coefficients. The metabolites that were significant are represented in blue, and those that were not significant in red. The developed models presented satisfactory performance (MDA: R^2^ adjusted from 0.5053 and estimated standard error was 0.1114; thiol: R^2^ adjusted from 0.5053, and estimated standard error of 0.1114).

**Figure 5:**
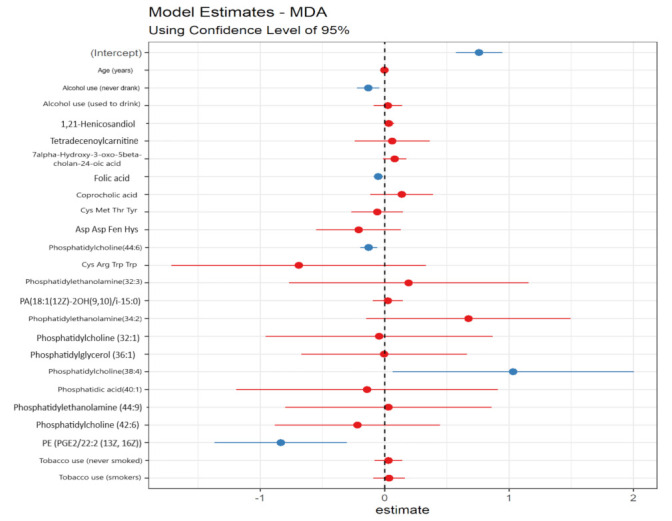

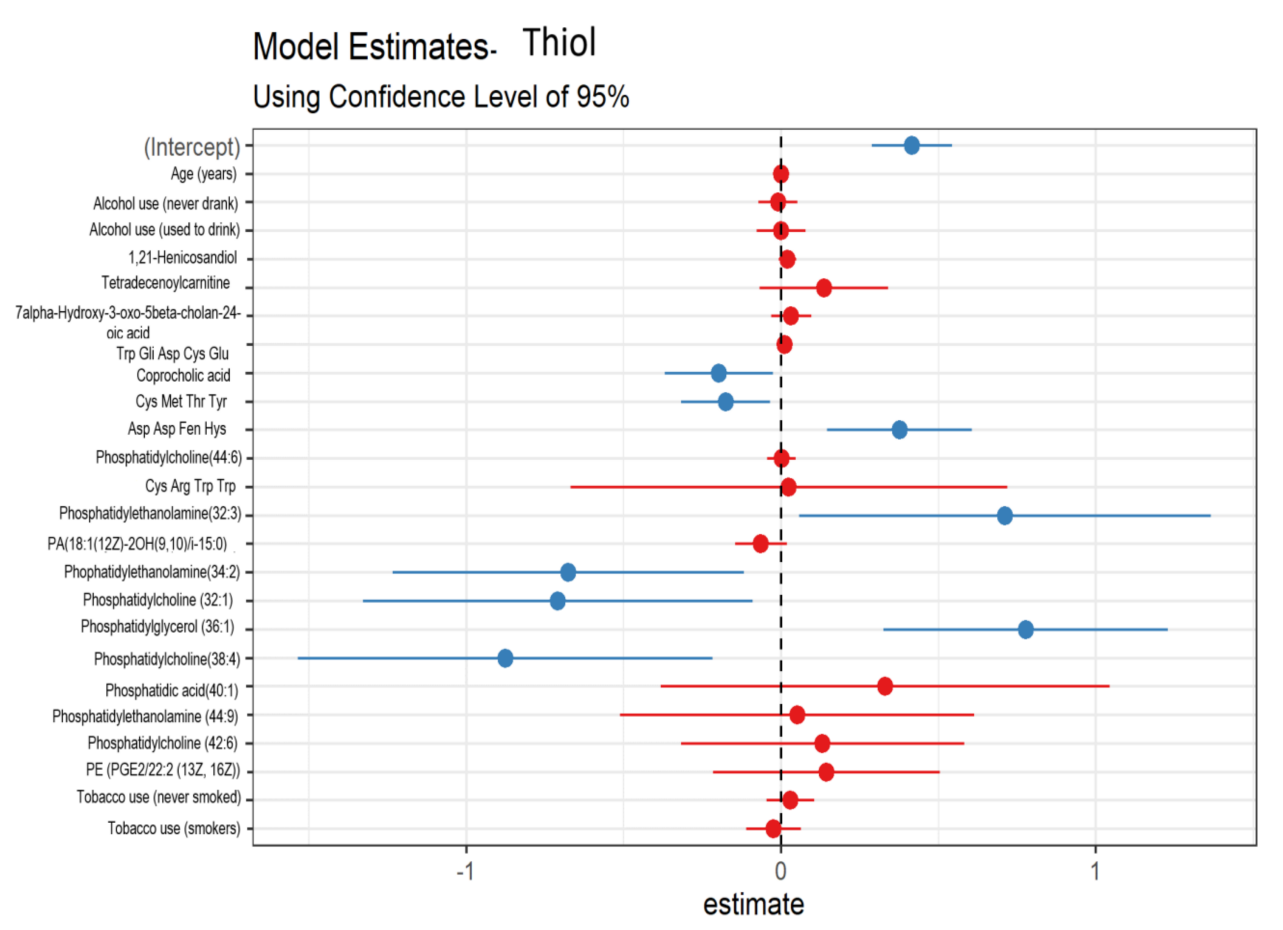

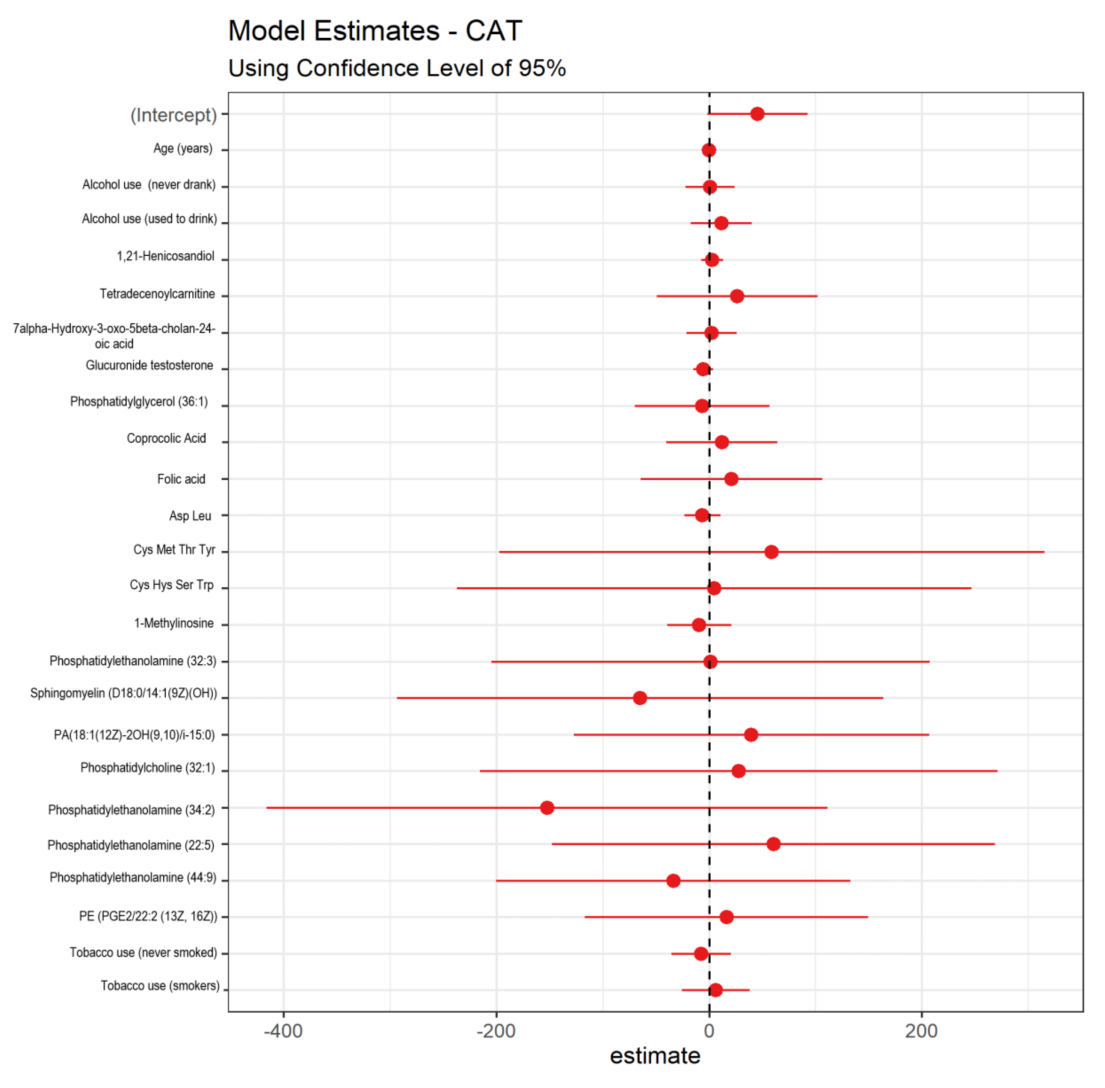

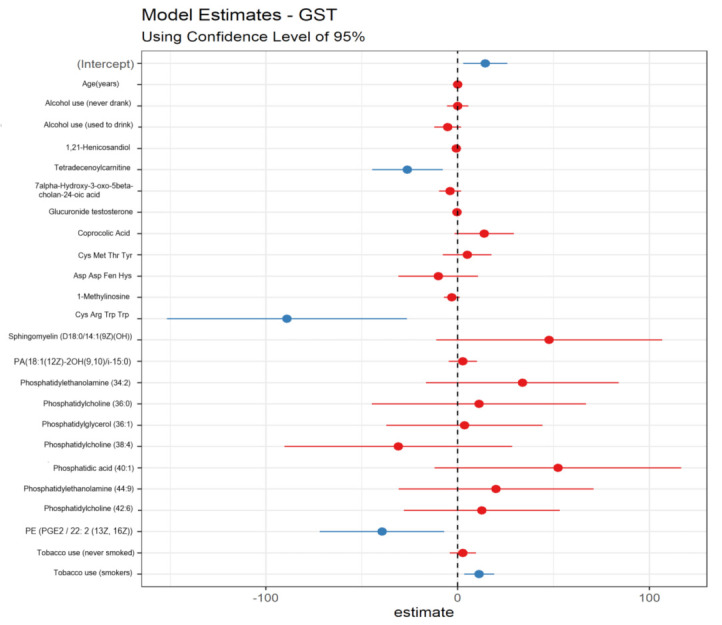
Multiple regression results and estimates of 𝛽0 and 𝛽κ for the main metabolites and sociodemographic variables for the biomarkers MDA, thiol, CAT, and GST. Note: Coefficients calculated using linear regression, with a 95% confidence interval (CI). In blue, significant urinary metabolites and in red, non-significant urinary metabolites.

Multiple regression models were used to assess the influence of demographic variables and metabolites on oxidative stress biomarkers (Figure 5). The results showed that there was a significant and negative influence (Beta < 0.00; *p* < 0.00) of the metabolites coprocholic acid, Cys Met Tre Tyr, phosphatidylethanolamine (34:2), phosphatidylcholine (32:1), and phosphatidylcholine (38:4) on thiol levels. Thus, with the increase in the concentration of these metabolites, a decrease in serum thiol concentration is expected. In contrast, the metabolites Cys Hys Ser Trp, phosphatidylethanolamine (32:3) and phosphatidylglycerol (36:1) had a significant and positive influence (Beta > 0.00) on the same biomarker. Thus, the increase in the concentration of these metabolites in the urine is accompanied by an increase in the serum thiol concentration. Phosphatidylcholine (44:6) and PE (PGE2/22:2 (13Z, 16Z)) had a significant negative effect (Beta < 0.00; *p* < 0.00) on the MDA biomarker. Phosphatidylcholine (38:4) had a positive (Beta > 0.00; *p* < 0.00) and significant influence on serum MDA levels.

No candidate metabolite proved to be significant to explain the variations of the CAT marker. On the other hand, the GST enzyme was significantly and negatively (Beta < 0.00; *p* < 0.00) influenced by tetradecenoylcarnitine, Cys Arg Trp Trp and PE (PGE2/22:2 (13Z, 16Z)). In addition, the smoking habit also influenced the levels of this enzyme, smokers with increased GST activity compared to former smokers. SOD enzyme activity was significantly and negatively influenced (Beta < 0.00 and *p* < 0.00) by phosphatidylcholine levels (44:6); and positively by 7alpha-hydroxy-3-oxo-5beta-cholan-24-oic acid (Beta > 0.00; *p* < 0.00).

Spearman’s correlation was used to verify the relationship between urinary metabolites and oxidative stress markers. The correlation coefficients and their significance are shown in Table 3. Many significant correlations were found (*p*-value < 0.05); however, the correlation coefficients showed low values, that is, close to zero.

The CAT biomarker showed low correlation with all metabolites. GST showed positive correlations with sphingomyelin (D18:0/14:1(9Z)(OH)), phosphatidylcholine (42:2), and phosphatidylcholine (44:6). On the other hand, 7alpha-hydroxy-3-oxo-5beta-cholan-24-oic acid, folic acid, and Trp Gly Asp Cys Glu were the most positively correlated with thiol. The biomarker MDA was negatively correlated (P^1^ < 0) with all urinary metabolites, except 1,21-henicosanediol. The highest correlations for this biomarker were observed with phenylalanylhydroxyproline, sphingomyelin (d18:1/16:0), Asp Leu, Trp Gly Asp Cys Glu, and tetrahydropteroyltri-L-glutamic acid. On the other hand, the SOD enzyme also showed negative correlations (P^1^ < 0) with all urinary metabolites, with the exception of 1,21-henicosanediol and was more negatively correlated with 1-methylinosine.

From these results, scatter plots were used to verify the behavior of the relationship between urinary metabolites and oxidative stress markers—thiol, MDA, CAT, SOD, and GST. The results showed that there is no linear correlation between them, therefore, the increase or decrease in the levels of oxidative stress markers does not imply a proportional change in urinary metabolites. Some correlations are shown in Figure S1.

### 3.4. Urinary Metabolites Associated with Chromosomal Aberrations

The relationship of discriminating urinary metabolites between groups of workers with environmental and occupational exposure to benzene, and chromosomal aberrations was also investigated using Spearman’s correlation. The following aberrations were considered: break rate, fragment rate, and metaphase rate with premature separation. The correlation coefficients and their significance are shown in Table 4. It is possible to verify that there were many significant correlations (*p*-value <0.05), but the correlation coefficients present low values, close to zero.

Breakage rate and fragment rate were negatively correlated with all urinary metabolites (P^1^ < 0). Phenylalanylhydroxyproline had the highest correlation with the breakage rate. Phenylalanylhydroxyproline, 1,21-henicosanediol, and Asp Leu showed higher correlations with the rate of fragments. Tetradecenoylcarnitine, 7alpha-hydroxy-3-oxo-5beta-cholan-24-oic acid, folic acid, Asp Leu, 1-methylinosine, and phosphatidylcholine (44:6) were the most positively correlated (P^1^ < 0) with the rate of premature metaphases.

To verify the behavior of the relationship between them, it was verified using scatter diagrams (Figure S2). The results obtained were similar to those observed in relation to oxidative stress markers. Therefore, the correlation between metabolites and chromosome breaks, fragment and metaphases presenting premature chromatid separation is not linear.

### 3.5. Potential Metabolomic Biomarkers Associated with Oxidative Damage and Benzene-Induced Chromosomal Aberrations

From the regression analyses, ten metabolites were significantly related to thiol, MDA e chromosomal aberrations. Thus, these were recognized as potential metabolomic biomarkers associated with oxidative effects induced by exposure to low concentrations of benzene. Their identity, as well as *p* values, fold change, and biochemical pathways in which they are biologically related is shown in Table 5.

The relationship between identified potential biomarkers and exposure time was investigated using Spearman’s Correlation. No significant correlation was observed (*p* > 0.05) (Table S5).

**Figure 6:**
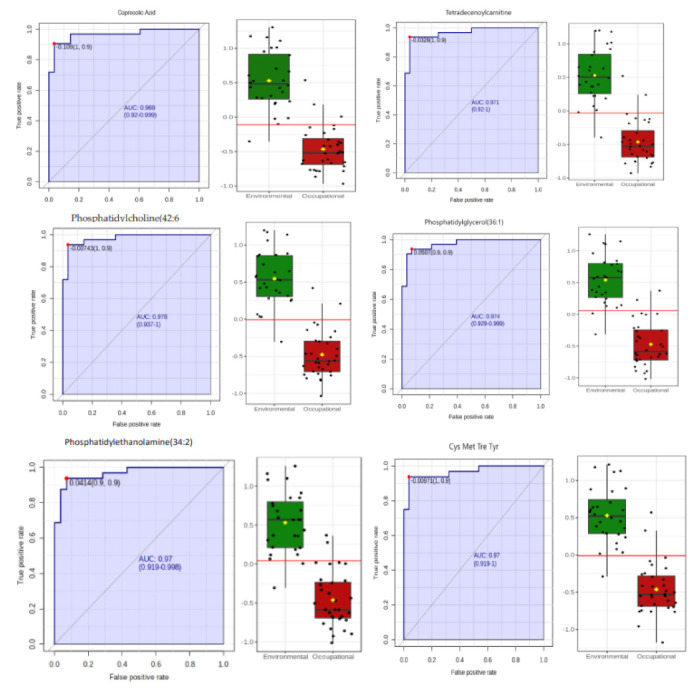
ROC curves of the discriminating urinary metabolites associated with oxidative stress and chromosomal aberrations induced by exposure to low concentrations of benzene. Note: On the left, the graphical representation of the calculated ROC curve with a 95% confidence interval for each biomarker candidate. AUC = area under the ROC curve. On the right, boxplot of metabolite concentrations in the environmental (green) and occupationally (red) exposed groups. A red horizontal line indicates the optimal cutoff point.

The ROC (receiver operating characteristic) curve was used to assess the performance of these potential early biomarkers in the assessment of toxicity induced by low concentrations of benzene. The ROC curve was used to evaluate the performance of these potential early biomarkers in the evaluation of toxicity induced by low concentrations of benzene. The ROC curve is widely used in the medical field, and consists of a graphical representation of the relationship between sensitivity and specificity of a diagnostic test. The area under the ROC curve (AUC) represents the global measure of a test’s ability to discriminate whether a specific condition is present or not. An AUC of 0.5 indicates that a test has no discriminating ability, while an AUC of 1.0 represents a test with perfect discrimination [36]. AUC > 0.85 is considered acceptable for clinical applications [37]. Based on the results of the ROC curve, all biomarker candidates performed satisfactorily, except for phenylalanylhydroxyproline and phosphatidylethanolamine (44:9) which had AUC <0.85. The ROC curve results of some potential biomarkers are shown in Figure 6.

### 3.6. Metabolic Pathway Analysis

**Figure 7:**
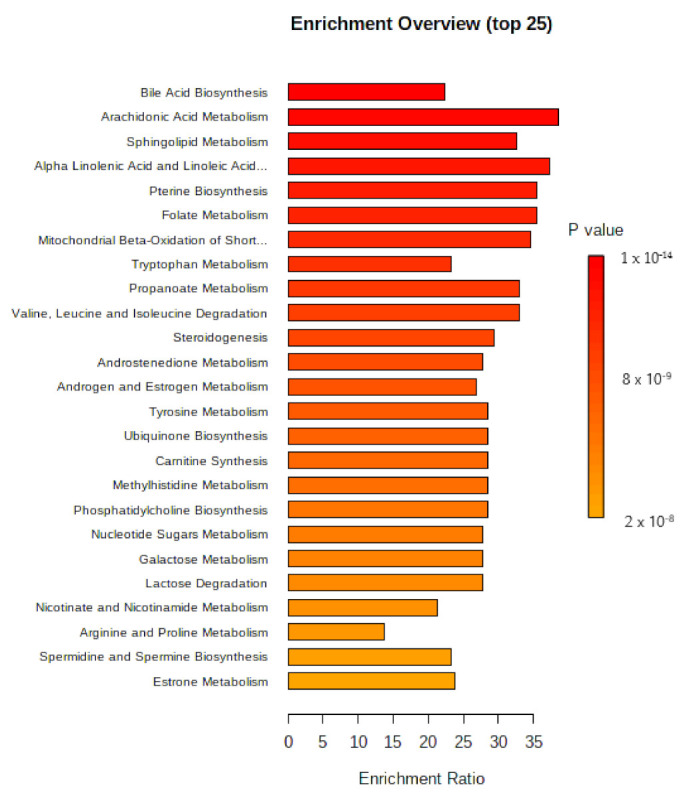
Bar graph with an overview of the analysis of enrichment of the metabolic 472 response against exposure to low concentrations of benzene.

From the identification of discriminant urinary metabolites identified in the PLS-DA model, we sought to identify the metabolic pathways involved in the biological response elicited by exposure to benzene. For this, the analysis of enrichment of the metabolic pathway was performed in Metaboanalyst^®^ 5.0 33. The results are presented in the form of bar graphs and Network View (Figure 7). It is observed that the metabolic alterations of greater impact (indicated in red) involve the bile acid synthesis pathways, lipid metabolism, amino acids, folate, mitochondrial beta-oxidation of short chain saturated fatty acids, and steroid hormone metabolism.

However, it is worth noting that the other biological pathways identified as related to benzene exposure are also relevant, given that the *p* values are quite low. This fact indicates that exposure to benzene, even at low concentrations, is capable of disturbing numerous biological pathways that are crucial for homeostasis, and their understanding is extremely important to understand the multiplicity of toxic effects induced by benzene.

### 3.7. Discussion

In this study, urine was chosen as a biofluid to investigate the metabolite profile of workers exposed both environmentally and occupationally to low concentrations of benzene in the air. It is a matrix traditionally used in biological monitoring studies because it has advantages such as: simple and non-invasive collection, allowing large volumes to be obtained and presenting a lower concentration of proteins compared to blood samples [38]. The urine samples were normalized by the concentration of urinary creatinine considering that the variation in the volume of urine can cause large variations in the concentration of the excreted metabolite leading to discrepancies on results [39].

The mean concentration of benzene in the atmospheric air of the fuel stations and gates was respectively 31.8 μg·m^3^ and 22.1 μg·m^3^. These results are below the technological reference value (TRV) of 3.19 mg·m^−3^, established by Brazilian legislation [40]. However, it is worth noting that exposure to low concentrations of benzene does not eliminate the risk; considering that there is no safe exposure limit to carcinogenic substances such as benzene [41]. This is corroborated by the results of this study, which proved that, even at low concentrations, benzene is capable of evoking metabolic disturbances, which were statistically associated with early toxic effects—oxidative stress and chromosomal alterations.

However, despite environmental monitoring having indicated similarity of exposure to hydrocarbons for workers in the gas station and ordinances, the analysis of the benzene concentration in air by percentile showed higher concentrations of xenobiotic in the 75th and 95th percentiles for the gas stations. This represents an oscillation in the chronic exposure to benzene during the working day, and indicates that there is a difference in the intensity of exposure during the performance of certain tasks, such as the aspiration of vapors when filling tanks.

In this study, the oxidative stress biomarkers—thiol and MDA were significantly different between groups, thus representing the toxic effects induced by chronic, low-intensity exposure to benzene. Oxidative stress is recognized as one of the toxic action mechanisms of benzene [42]. Oxidative stress represents a state of imbalance between the generation of reactive species and the body’s antioxidant capacity. These reactive oxygen species (ROS), such as superoxide anion, hydrogen peroxide, hydroxyl radical, are unstable and highly reactive. They are produced through the redox cleavage of reactive products originated during the biotransformation of benzene, especially hydroquinone and benzoquinone [43]. Scientific literature indicates that such reactive species can damage biomolecules such as lipids, proteins, and genetic material [44,45,46]. Many chronic diseases such as type 2 diabetes mellitus, cardiovascular disease, Alzheimer’s disease, various types of cancer (leukemia, lung, liver, etc.,) and chronic inflammation are associated with oxidative stress. It is known that continuous oxidative stress can elicit inflammatory mechanisms, with synthesis and secretion of pro-inflammatory cytokines [47].

Inflammation is a physiological process of body protection, triggered in situations that can cause damage, such as infections by microorganisms, exposure to allergens, ionizing radiation and toxic substances. Inflammation has two stages—acute and chronic. Acute inflammation is mediated by innate immunity, has a short duration and usually results in a beneficial effect on the body. On the other hand, chronic inflammation, which lasts for a long time, is mediated by leukocytes (mast cells and monocytes) that migrate to the site of damage, where they promote an increase in oxygen uptake (respiratory explosion) with a consequent increase in the local production of ROS; production of arachidonic acid derivatives and also secretion of cytokines and chemokines; which recruit more inflammatory cells to the site and once again increase the production of ROS [48]. It should be noted that chronic inflammation is associated with a higher risk of various types of cancer [49]. Thus, there is an important relationship between oxidative stress and inflammation.

Workers occupationally exposed to benzene analyzed in this study had higher counts of monocytes and basophils in peripheral blood (leukocytes with an important role in inflammation) [29], in addition to prostaglandin PE (PGE2/22:2 (13Z, 16Z)), leukotrinene D5 and oxidized lipids were identified among the most discriminating urinary metabolites among the analyzed groups. These results indicate that oxidative stress and inflammatory state are the most relevant mechanisms of toxic action in exposure to low concentrations of benzene.

This subtle difference in exposure was not detected, for example, through exposure biomarkers—A*tt*M and *S*PMA; which did not show significant difference between groups. In this context, metabolomics presents itself as a promising, sensitive, and appropriate tool to assess the risks arising from exposure to benzene. Analysis supervised by PLS-DA and OPLS-DA was able to recognize this subtle difference, thus indicating that even at low doses, the biological response induced by exposure to this hydrocarbon was different between groups.

Metabolomic analysis can offer simultaneous analysis of a large number of metabolites without any prior knowledge of these compounds [50]. However, this comprehensive analysis is not an easy task. As the metabolome is made up of an immense amount of metabolites belonging to different chemical classes such as amino acids, carbohydrates, nucleotides, and lipids that have a wide range of physicochemical properties and are present in a wide range of concentration [51,52], the analysis of the metabolic profile represents a great challenge in the field of analytical chemistry. In the field of environmental and occupational toxicology, it represents a promising tool for a holistic understanding of the interaction between chemicals and the human organism.

The validation of the developed PLS-DA and OPLS-DA models is important to confirm the quality of the developed models and attest to their predictive capacity. The obtained R^2^ explains the model’s variance and gives information about the goodness of fit, while the Q^2^ predicts the variance and provides information about the model’s predictability. A robust PLS-DA model should have high values of R^2^ and Q^2^ and not differ by more than 0.2–0.3 from each other [53,54]. Model validation is also useful in identifying the optimal number of latent variables needed to describe the entire data variance; and avoid under- or over-adjustment issues. According to Godzien et al., (2013) [55], the usual values of these coefficients for biological experiments are Q^2^ > 0.4 and R^2^ > 0.7.

Chemical exposure leads to alterations in metabolism and/or gene expression (deletion or overexpression) and such alteration may represent a specific pattern, called metabolic signature [56]. This metabolic signature may reflect this exposure, which has supported the use of metabolomics in the assessment of exposure to environmental and occupational chemical agents. According to Wishart (2016) [57], metabolites are more representative of the phenotype, as they change more quickly than genetic material and, therefore, indicate current biological events. It is noteworthy that the metabolome is compartmental, dynamic, and related to the phenotype. Thus, its assessment represents a holistic approach to the individual’s physiological and/or pathological state, since it integrates genetic and environmental factors [15,16,58]. Thus, the wealth of information acquired in undirected metabolomics is an excellent tool for a better understanding of the organism’s biochemical events in environmental exposure to chemical agents.

Therefore, the results observed in this study demonstrated that the exposure of individuals to benzene, even at low doses, was capable of causing metabolic changes in urine, which were identified through non-directed metabolomics. Due to the mildness of the alterations, including the differences between the two groups, they were only identified due to the high sensitivity of the UHPLC-ESI-Q-TOF-MS, demonstrating its robustness and capacity for wide coverage of the urinary metabolome. This justifies its choice for application in environmental and occupational metabolomics studies of chemical contaminants in low concentrations.

The discriminating metabolites significantly correlated with oxidative stress and benzene-induced chromosomal aberrations act predominantly in biological pathways related to lipid and amino acid synthesis, metabolism, and peroxidation (Table 5).

Studies on urine, plasma, and bone marrow metabolomics in C3H/He-exposed rats were conducted by Sun et al., (2012) [59] and Sun et al., (2014) [60], respectively. In urine, the metabolic disturbances reported involved the metabolism pathways of purines, spermidines, fatty acids, tryptophan, and peptides [59]. In plasma, L-acetylcarnitine, p-coumaric acid, L-tyrosine, L-phenylalanine and lysine were significantly altered; while 5-hydroxyindoleacetic acid, histamine, L-histidine, N-methylhistamine, L-acetylcarnitine, pyrrolidone carboxylic acid, and palmitoylcarnitine in bone marrow [60]. In another investigation also conducted in an animal model, Yu et al. (2021) [61] investigated the lipidome of bone marrow cells from male C57BL/6 rats, exposed to 150 mg/kg of benzene using LC-MS/MS technology, in order to understand the mechanisms associated with benzene hematotoxicity. The results showed significant changes in the levels of glycerophospholipids, sphingolipids, linoleic acid metabolism, amino acids, and unsaturated fatty acid biosynthesis. Based on this, the authors hypothesized that disturbances in the glycerophospholipid pathway affect autophagy, while the sphingolipid pathway seems to influence proliferation and apoptosis; and both processes play an important role in benzene-induced hematopoietic toxicity [61].

In this study, the metabolism of several classes of lipids was associated with exposure to benzene, including the biosynthesis of bile acids. Coprolic acid, taurocholic acid, 3-chenodeoxycholic sulfate, 3-oxocholic acid, 7b-hydroxy-3-oxo-5b-cholanoic acid, nutriacolic acid, and hodeoxycholic acid were excreted in smaller amounts in the urine of gas station workers. Bile acids are produced in the liver from cholesterol and later converted to bile salts by conjugation with glycine or taurine. They are then stored in the gallbladder and subsequently excreted in the bile, along with its other components, in the duodenum, where they are deconjugated and reduced to urobilinogens by the action of the microbiota. These can be excreted in the feces, partially reabsorbed and about 5% excreted in the urine. Biologically, they play an important role in the intestinal absorption of lipophilic substances, participate in the regulation of cholesterol and in the metabolism of lipids and carbohydrates, stimulate bile flow, act as cellular signals, in the excretion of toxic substances, in the maintenance of a healthy organism, gut microbiota, and innate immunity [62,63]. Sun et al., (2020) [64] reported changes in the metabolic profile of the cecal content of mice exposed to benzene for 30 days. Changes were mainly observed in bile acids, fatty acids, carboxylic acids and derivatives, glycerolipids, glycerophospholipids, prenole lipids, steroids, and steroid derivatives. These results indicate that benzene is capable of causing intestinal dysbiosis with significant changes in several species, such as Proteobacteria, Bacteroidetes, and Actinobacteria, which are involved in the biotransformation of bile acids [65].

It is believed that the disturbance in bile acid excretion may also be due to the liver toxicity of benzene. Reactive metabolites formed in the liver during its biotransformation can influence the regulatory pathways of bile acid biosynthesis. Occupationally exposed individuals had higher values for liver function markers—aspartate aminotransferase (AST) and direct bilirubin. AST is an enzyme that catalyzes the interconversion of amino acids to 2-oxo-acids through the transfer of amino groups, found mainly in the heart and liver, cytoplasm, and mitochondria. Direct bilirubin, on the other hand, is a biotransformation product of the heme group, which is conjugated to glucuronic acid in the liver. Direct hyperbilirubinemia is associated with hepatocyte damage or biliary obstruction [66]. Other studies corroborate these results. In the investigation conducted by Moro et al., (2017) [67], albumin and TGO were significantly higher in gas station attendants compared to the control group. Andrea and Reddy, (2014) [68] detected higher values of liver enzyme alkaline phosphatase (ALP), aspartate amino transferase (AST), and alanine amino transferase in children accidentally exposed to benzene after a burning incident at the British Oil Refinery (BP) in the city of Texas. Additionally, the intestinal reabsorption of bile acids can be compromised by benzene-induced intestinal dysbiosis.

Koelmel et al. (2020) [14] state that lipids are ubiquitous metabolites that play different physiological roles, and are especially involved in biological processes such as oxidative stress, inflammation, obesity and endocrine disruption. As many environmental stressors exert their toxic effects through disturbances in these biological processes, the investigation of changes in lipid metabolism represents an important strategy to elucidate mechanisms of toxic action, as well as to identify new biomarkers of exposure to such stressors. Disturbances in lipid metabolism have been related to several pathologies [69,70,71,72,73].

Arachidonic acid metabolism was also implicated in the biological response of workers exposed to benzene investigated in this study. Previous studies have shown that the metabolism of this group of lipids is involved in benzene myelotoxicity [74,75]. Prostaglandins are prostanoids synthesized from arachidonic acid by cyclooxygenase enzymes (COX-1 and COX-2), and play physiological and pro-inflammatory functions. Prostanoids are often implicated in the initiation of carcinogenesis, representing the link between inflammation and cancer [76]. The increase in PGE2 was related to arachidonic acid peroxidation, increased phagocytic activity of macrophages, and decreased cellularity in the bone marrow [74,75]. In this study, a reduction in PGE1 and an increase in PGF2 in the urine of occupationally exposed workers were observed. PGE1 is a prostanoid that plays important physiological roles such as vasodilation, inhibitor of platelet aggregation, mediator of inflammation, and even hepatoprotective action. Animal studies have demonstrated hepatic cytoprotection of PGE1 through inhibition of T-cell mediated cytotoxicity, increased DNA synthesis, cyclic AMP, and liver tissue ATP levels [77,78,79]. The PGF2 is one of the most common protaglandins, is produced in many organs, and has a wide range of effects. Pabst et al. (2017) [73] reported an increase in this prostaglandin in plasma and bone marrow of patients with acute myeloid leukemia (AML).

Another metabolic pathway associated with early toxic effects induced by benzene was the metabolism of sphingolipids. These are physiologically implicated in the regulation of cell growth, differentiation, and apoptosis [80]. Among the types of sphingolipids, ceramides, structural membrane components, and secondary messengers in cell signaling have been implicated in the pathophysiology of diseases such as diabetes, cancer, Alzheimer’s disease, multiple sclerosis, and others [70]. In our study, sphingomyelin (d18:0/14:1(9Z)(OH)) and sphingomyelin (d18:1/16:0) decreased in the urine of occupationally exposed workers and was significantly associated with early toxic effects induced by benzene. Decline in plasma sphingolipids has also been described in carriers and AML [73,81]. A similar result was found by Robinson et al., (2021) [81], the authors investigated the relationship between reactive oxygen species (ROS) and the metabolome in AML cells. Sphingolipids were significantly decreased. Based on these findings, the authors postulated that ROS are important in regulating the synthesis and/or degradation of sphingolipids.

Polyunsaturated fatty acids have also been implicated in benzene-induced toxicity. In our study, in addition to fatty acids, fatty acid amides (oleamide, linoleamide and palmitoleamide) and oxylipin 14,15-DiHETrE were also detected in the urine of workers exposed to benzene, and were significantly reduced.

These lipids are considered essential and play an important role in membrane maintenance, signal transduction, and anti-inflammatory properties. Musharraf et al., (2016) [82] investigated the serum metabolome of individuals with AML, acute lymphocytic leukemia, and aplastic anemia using the CG-MS technique. The authors reported that palmitic acid was significantly reduced in patients with ALL and AML. Stearic and oleic acids, on the other hand, had higher concentrations in patients with these diseases [82]. It has been shown that AML patients have an overproduction of reactive oxygen species (ROS), and that these produce changes in carbohydrate metabolism, sphingolipids, fatty acid oxidation, purine metabolism, and amino acid homeostasis [81]. The α-linoleic acid (ALA), a polyunsaturated fatty acid, has an anti-inflammatory action due to its bioconversion into long-chain polyunsaturated fatty acids and, later, into oxylipins, bioactive lipid mediators [73]. Pabst et al., (2017) [73] reported an increase in fatty amides (oleamide and palmitoleoyl ethanolamide) in AML cells. A oleamide is an endogenous bioactive signaling molecule, similar to endocannabinoids, which participates in sleep regulation via the CB1 receptor and has anti-inflammatory action [83,84]. It is believed that the reduction of these bioactive metabolites in the urine of occupationally exposed workers is a consequence of their increased consumption, an adaptive response triggered in order to contain the toxic effects induced by benzene.

It should be noted that folate, also identified as an important pathway related to benzene exposure, is necessary for cell division and DNA synthesis; and according to Morgan and Smith (2002) [85], folate metabolism is associated with the maintenance and integrity of DNA. Folate insufficiency can lead to anemia, neutropenia, and pancytopenia [85].

Metabolomics also identified tryptophan metabolites—indoleacetic acid, 5-hydroxyindoleacetic acid and 5-hydroxy-6-methoxyindole, all of which had VIP >1. Urine indoleacetic acid was identified as a potential biomarker of benzene-induced toxicity in mice in the study by Sun et al., (2012) [59]. Evidence indicates that indoleacetic acid activates the aryl hydrocarbon receptor (AhR) and positively regulates cytochrome P-450 enzymes, implicated in tumorigenesis and cancer proliferation [86].

The propanoate metabolism and amino acid degradation of valine, leucine, and isoleucine were also listed among the most important pathways in the biological response to benzene exposure. These pathways are believed to be consequences of protein oxidation. Propanate is a short-chain fatty acid formed during the catabolism of branched-chain amino acids and methionine, and its concentration in plasma increases in amino acid oxidation states [87]. The reactive species produced during benzene bioactivation are able to form adducts with albumin, hemoglobin, and bone marrow proteins. Spatari et al., (2012) [88] found higher levels of advanced protein oxidation products in the serum of workers at an oil refinery located in Sicily, exposed to low concentrations of benzene (70.14 ± 69.71 μg/m^3^). Avery, (2011) [89] states that the susceptibility of proteins to oxidation depends on the relative content of oxidation-sensitive amino acid residues, the presence of metal-binding sites, location of proteins in the cell, molecular conformation, and rate of degradation. Methionine is one of the amino acid residues most prone to oxidation, and almost all organisms express methionine sulfoxide reductase enzymes to reverse this change. Oxidized cysteine and tryptophan residues are other useful markers of oxidation-modified proteins [89].

Based on the developed PLS-DA and OPLS-DA models, 38 metabolites in the urine responsible for discrimination between groups of workers exposed to benzene were identified. It is observed that they are metabolites belonging to the classes of peptides, carnitines, and predominantly lipids (Table 2).

Changes in lipid metabolism and increased oxidative stress resulting from exposure to benzene have been reported in recent studies. Rothman et al. (2021) [90] investigated the urinary metabolome of Chinese workers exposed to high concentrations of benzene (weighted average exposure of 8 h; 20 ppm), using liquid chromatography and high-resolution Fourier transform mass spectrometry (HRMS). The results showed that exposure to benzene leads to alterations in carnitine transport, fatty acid metabolism, sulfur amino acid metabolism, glycolysis, gluconeogenesis, and branched-chain amino acid metabolism. Mono- and polyunsaturated and short-chain fatty acids are also significantly associated with S-phenylmercapturic acid and leukocytes, and reported as key mediators of benzene-induced hematotoxicity in the study by Guo et al. (2022) [91].

The β-oxidation of fatty acids is a crucial pathway for hematopoietic stem cells (HSCs) and leukemic cells. Sun et al., (2014) [60] demonstrated that exposure to benzene induced disturbances in the levels of metabolites of the fatty acid β-oxidation pathway in the bone marrow of C3H/He mice. In another later study, Sun et al., (2016) [92] identified that exposure to benzene resulted in abnormal transport of fatty acids and β-oxidation of these lipids in bone marrow cells from C3H/He mice. It is worth noting that carnitine transport and fatty acid metabolism are involved in mitochondrial function.

L-carnitine (LC) is responsible for the transport of fatty acids across the mitochondrial membrane, and its levels were significantly reduced in animals treated with benzene. Mitochondrial dysfunction has been reported with a significant reduction in ATP levels and membrane potential. Furthermore, an increase in the generation of ROS and H_2_O_2_ was also observed, which led to oxidative stress and lipid peroxidation [92]. The disturbance in the β-oxidation of fatty acids decreased the production of NADPH, an important mitochondrial antioxidant, as well as the levels of ATP. These findings indicated that benzene induces mitochondrial dysfunction and oxidative stress, which may be one of the causative mechanisms of hematotoxicity [67,90]. In a recent study, Sun, Man, et al., (2020) [93] demonstrated that co-treatment with L-carnitine in K562 erythroleukemia cells treated with 1,4-benzoquinone, reduces the rate of apoptosis, DNA damage, reduces oxidative stress and promotes the β-oxidation of fatty acids.

Ten metabolites showed significant, low and non-linear correlations with biomarkers of oxidative stress and chromosomal aberrations (Table 3 and Table 4). These have been recognized as potential early-effect metabolomic biomarkers, induced by exposure to low concentrations of benzene. All of them are involved in metabolic pathways associated with the synthesis and metabolism of lipids and fatty acids. Based on these results, we speculate that oxidative damage is especially related to disturbances in lipid metabolism and transport. These results represent relevant information to guide future investigations into the mechanism underlying benzene-induced oxidative damage.

### 3.8. Limitations of the Study

Among the limitations of this study, the small sample size (*n* = 60) stands out, which may have been a source of random variation in the results of the two groups analyzed. The cross-sectional approach of the study also represents a limitation, considering that in this type of study, exposure and effect are evaluated concomitantly. Furthermore, the intensities of exposure to benzene in both groups of workers (environmental and occupational) were similar. Finally, the identification of metabolites was putatively, according to level 2, recognized by the Metabolomics Standards Initiative (MSI).

## 4. Conclusions

The study of metabolomic using the UHPLC-ESI-Q-TOF-MS technique revealed metabolic disturbances in workers exposed to low concentrations of benzene. Despite the history of low exposure to hydrocarbons for both groups, the intensity of exposure among workers at gas stations is greater during operations that involve direct contact with fuel.

The disturbances identified involved several metabolic pathways, with emphasis on the metabolism and transport of lipids and fatty acids. Ten urinary metabolites showed significant correlations, low and non-linear, with biomarkers of oxidative stress and chromosomal aberrations—toxic effects induced by benzene in the participants of this study, previously identified. Thus, the determination of the ten urinary metabolites can be used for a more comprehensive and accurate assessment of benzene-induced cytotoxicity and genotoxicity.

It is noteworthy that this study has advantages and limitations. Among the advantages is the unprecedented association established between chronic exposure to low doses of benzene and alterations in the urinary metabolome. On the other hand, the main limitation refers to the small size of the studied population. Furthermore, the unambiguous identification of urinary metabolites by comparing spectra with analytical standards is also necessary. Therefore, the ability of potential biomarkers to predict health outcomes needs to be confirmed through future and similar studies in a larger population.

Additionally, this study proves the power of metabolomics to provide relevant information to understand the biological response to exposure to low concentrations of xenobiotics and identification of early effect biomarkers.

## Figures and Tables

**Table 1:** Gradient elution and rate flow used in the chromatographic separation.

Time (min)	Rate (mL/min)	% A	% B
0	0.200	96.0	4.0
2	0.200	96.0	4.0
7	0.200	81.7	18.3
12	0.223	50.0	50
14	0.400	0.1	99.9
16	0.480	0.1	99.9
19	0.480	96.0	4.0
19.10	0.200	96.0	4.0
20	0.200	96.0	4.0

**Table 2:** Identification of urinary metabolic biomarkers related to exposure to low concentrations of benzene.

Metabolites	M	VIP ^a^ Score	*p* ^b^ Values	Chemical Category
Phenylalanylhydroxyproline	279.13	2.42	0.0000	Peptide
Tetrahydropteroyltri-L glutamic acid	703.25	2.29	0.0001	Tetrahydrofolic acid
Trp Gln Asp Cys Glu	679.22	2.22	0.0000	Peptide
Testosterone glucuronide	465.24	2.19	0.0000	Steroid glucuronide
7alpha-Hydroxy-3-oxo-5beta-cholan-24-oic acid	390.27	2.18	0.0000	Bile acid
Phosphatidylcholine(44:6)	889.65	2.15	0.0000	Phosphatidylcholine
Asp Asp Phe Hys	532.19	2.14	0.0000	Peptide
Phosphatidylcholine(42:6)	861.62	2.11	00000	Glycerophospholipid
Phosphatidylcholine(42:2)	869.68	2.10	0.0000	Glycerophospholipid
Phosphatidylcholine(22:2)	825.66	2.10	0.0000	Glycerophospholipid
Folic acid	441.13	2.10	0.0000	Pterins
Phosphatidylethanolamine PGE2/22:2(13Z,16Z)	867.56	2.10	0.0000	Glycerophospholipids
Phosphatidylcholine(40:3)	891.64	2.10	0.0000	Phosphatidylcholine
Cys Hys Ser Trp	531.19	2.10	0.0000	Peptide
1,21-Henicosanediol	328.33	2.09	0.0000	Long chain fatty alcohol
Heptadecanoic carnitine	413.35	2.08	0.0000	Acyl carnitine
Phosphatidylglycerol (36:1)	776.55	2.06	0.0000	Phosphatidylglycerol
Phophatidylethanolamine(44:9)	841.56	2.06	0.0000	Phosphatidylethanolamine
1-(9Z-heptadecenoyl)-2-(7Z, 10Z, 13Z, 16Z-docosatetraenoyl) -glycero-3-phosphoserine	823.53	2.06	0.0000	Glycerophospholipids
1-Methylinosine	283.28	2.05	0.0001	Purine nucleoside
Cys Met Thr Tyr	517.85	2.05	0.0000	Peptide
Tetradecenoylcarnitine	369.28	2.05	0.0000	Acyl carnitine
Phosphatidylethanolamine(22:5)	778.09	2.05	0.0000	Glycerophospholipids
Phosphatidylcholine(36:0)	775.64	2.05	0.0000	Glycerophospholipids
Coprocholic acid	450.33	2.04	0.0000	Bile acid
Asp Leu	494.24	2.04	0.0000	Peptide
Phosphatidylserine(38:1)	817.58	2.04	0.0000	Phosphatidylserine
Phosphatidic acid(40:1)	758.58	2.03	0.0000	1,2-diacylglycerol-3-phosphate
Phosphatidic acid PA(18:1(12Z)-2OH(9,10)/i-15:0)	692.46	2.03	0.0000	Glycerophospholipids
Phosphatidylcholine(32:1)	731.54	2.02	0.0000	Glycerophospholipids
Phosphatidylcholine(34:1)	759.57	2.02	0.0000	Glycerophospholipids
Phosphatidylethanolamine(34:2)	715.51	2.02	0.0000	Phosphatidylethanolamine
Cys Arg Trp Trp	649.27	2.01	0.0000	Peptide
Sphingomyelin (D18: 0/14: 1 (9Z) (OH))	688.51	2.01	0.0000	Sphingolipid
Sphingomyelin (d18:1/16:0)	702.56	2.01	0.0000	Sphingolipid
Phosphatidylcholine(38:4)	795.61	2.01	0.0000	Glycerophosphocholine
Phosphatidylethanolamine(35:0)	733.56	2.01	0.0000	Glycerophospholipids
Phosphatidylethanolamine(32:3)	671.48	2.00	0.0000	Glycerophosphoethanolamine

Note: M: average molecular weight. ^a^: VIP score was obtained from the PLS-DA model. ^b^: *p*-values were calculated from nonparametric Mann—Whitney test between occupational and environmental exposure groups. Thirty-eight significantly changed (VIP > 2, *p* value < 0.05 and FDR < 0.01) endogenous metabolites related to benzene exposure are listed in this table.

**Table 3:** Spearman correlation between urinary metabolites and oxidative stress markers.

	CAT		GST		THIOL		MDA		SOD	
	P¹	*p* Value	P¹	*p* Value	P¹	*p* Value	P¹	*p* Value	P¹	*p* Value
Phenylalanylhydroxyproline	−0.068	0.605	0.205	0.116	0.083	0.531	−0.342	0.007	−0.012	0.928
Sphingomyelin (d18:1/16:0)	−0.019	0.887	0.162	0.218	0.149	0.256	−0.356	0.005	−0.002	0.987
1,21-Henicosanediol	0.008	0.949	−0.017	0.897	0.047	0.721	0.007	0.957	0.007	0.960
Tetradecenoylcarnitine	0.114	0.386	0.264	0.041	0.314	0.014	−0.272	0.036	−0.215	0.099
7alpha-Hydroxy-3-oxo-5beta-cholan-24-oic acid	0.091	0.490	0.275	0.033	0.333	0.009	−0.216	0.098	−0.183	0.161
Testosterone glucuronide	0.034	0.796	0.268	0.039	0.273	0.035	−0.306	0.017	−0.267	0.039
Phosphatidylglycerol (36:1)	−0.146	0.267	−0.079	0.548	0.153	0.242	−0.083	0.528	0.274	0.034
Coprocholic acid	0.135	0.303	0.302	0.019	0.238	0.067	−0.305	0.018	−0.280	0.030
Folic acid	0.064	0.627	0.281	0.030	0.331	0.010	−0.276	0.033	−0.251	0.053
Asp Leu	0.097	0.459	0.246	0.058	0.236	0.069	−0.310	0.016	−0.223	0.087
Cys Met Thr Tyr	0.141	0.282	0.266	0.040	0.214	0.100	−0.303	0.019	−0.236	0.070
1-(9Z-heptadecenoyl)-2-(7Z,10Z, 13Z, 16Z-docosatetraenoyl)-glycero-3-phosphoserine	0.086	0.514	0.295	0.022	0.281	0.030	−0.275	0.034	−0.260	0.045
Cys Hys Ser Trp	0.030	0.817	0.275	0.033	0.254	0.051	−0.248	0.056	−0.261	0.044
Asp Asp Fen Hys	0.054	0.680	0.287	0.026	0.283	0.028	−0.246	0.059	−0.275	0.034
Cys Arg Trp Trp	0.113	0.391	0.297	0.021	0.291	0.024	−0.265	0.041	−0.262	0.043
Phosphatidylethanolamine(32:3)	0.103	0.433	0.308	0.017	0.303	0.019	−0.265	0.041	−0.264	0.042
Sphingomyelin (D18: 0/14: 1 (9Z) (OH))	0.087	0.511	0.312	0.015	0.284	0.028	−0.256	0.049	−0.270	0.037
Trp Gln Asp Cys Glu	0.039	0.770	0.294	0.022	0.321	0.012	−0.330	0.010	−0.213	0.103
Tetrahydropteroyltri-L-glutamic acid	−0.005	0.969	0.300	0.020	0.240	0.065	−0.348	0.006	−0.278	0.031
Phosphatidic acid PA(18:1(12Z)-2OH(9,10)/i-15:0)	0.095	0.472	0.304	0.018	0.268	0.038	−0.253	0.051	−0.256	0.048
Phosphatidylcholine(32:1)	0.096	0.464	0.306	0.017	0.286	0.027	−0.258	0.047	−0.259	0.045
Phosphatidylethanolamine(34:2)	0.107	0.414	0.295	0.022	0.277	0.032	−0.256	0.049	−0.261	0.044
1-Methylinosine	0.044	0.739	0.292	0.024	0.247	0.057	−0.196	0.132	−0.401	0.002
Phosphatidylethanolamine(35:0)	0.121	0.358	0.291	0.024	0.274	0.034	−0.253	0.051	−0.251	0.053
Heptadecanoic carnitine	0.064	0.625	0.301	0.019	0.290	0.025	−0.266	0.040	−0.252	0.052
Phosphatidylcholine(36:0)	0.081	0.539	0.293	0.023	0.291	0.024	−0.269	0.038	−0.262	0.043
Phophatidylethanolamine(22:5)	0.096	0.468	0.297	0.021	0.268	0.038	−0.255	0.049	−0.279	0.031
Phosphatidylcholine(38:4)	0.091	0.489	0.308	0.017	0.266	0.040	−0.256	0.048	−0.251	0.053
Phosphatidic acid(40:1)	0.090	0.493	0.299	0.021	0.287	0.026	−0.273	0.035	−0.253	0.051
Phosphatidylcholine(34:1)	0.093	0.480	0.295	0.022	0.275	0.034	−0.261	0.044	−0.262	0.043
Phosphatidylserine(38:1)	0.088	0.503	0.296	0.022	0.277	0.032	−0.259	0.045	−0.252	0.052
Phophatidylethanolamine(44:9)	0.089	0.501	0.304	0.018	0.294	0.023	−0.262	0.043	−0.262	0.043
Phosphatidylcholine(22:2)	0.100	0.448	0.299	0.020	0.289	0.025	−0.256	0.048	−0.242	0.063
Phosphatidylcholine(42:6)	0.082	0.536	0.288	0.026	0.288	0.026	−0.261	0.044	−0.257	0.048
Phosphatidylcholine(40:3)	0.113	0.391	0.297	0.021	0.291	0.024	−0.265	0.041	−0.262	0.043
Phosphatidylethanolamine PGE2/22:2(13Z, 16Z)	0.095	0.473	0.289	0.025	0.263	0.043	−0.281	0.029	−0.238	0.067
Phosphatidylcholine(42:2)	0.079	0.547	0.311	0.015	0.285	0.027	−0.274	0.034	−0.279	0.031
Phosphatidylcholine(44:6)	0.075	0.569	0.310	0.016	0.293	0.023	−0.270	0.037	−0.252	0.052

P¹: Spearman’s correlation coefficient; *p*-values were calculated from nonparametric Mann—Whitney test.

**Table 4:** Spearman correlation between urinary metabolites and chromosomal aberrations.

	Break Rate		Fragments Rate		Metaphase Rate with Premature Separation	
	P¹	*p* Value	P¹	*p* Value	P¹	*p* Value
Phenylalanylhydroxyproline	−0.315	0.014	−0.316	0.014	0.196	0.136
Sphingomyelin (d18:1/16:0)	−0.271	0.036	−0.263	0.042	0.150	0.256
1,21-Henicosanediol	−0.334	0.009	−0.331	0.010	0.154	0.243
Tetradecenoylcarnitine	−0.291	0.024	−0.290	0.024	0.320	0.013
7alpha-Hydroxy-3-oxo-5beta-cholan-24-oic acid	−0.244	0.060	−0.249	0.055	0.353	0.006
Testosterone glucuronide	−0.272	0.035	−0.272	0.035	0.300	0.021
Phosphatidylglycerol (36:1)	0083	0.528	0.089	0.498	0.130	0.327
Coprocholic acid	−0.287	0.026	−0.298	0.021	0.275	0.035
Folic acid	−0.241	0.063	−0.243	0.061	0.349	0.007
Asp Leu	−0.303	0.019	−0.308	0.017	0.319	0.014
Cys Met Thr Tyr	−0.315	0.014	−0.313	0.015	0.269	0.039
1-(9Z-heptadecenoyl)-2-(7Z,10Z, 13Z, 16Z-docosatetraenoyl)-glycero-3-phosphoserine	−0.282	0.029	−0.290	0.025	0.304	0.019
Cys Hys Ser Trp	−0.258	0.047	−0.250	0.054	0.281	0.031
Asp Asp Fen Hys	−0.279	0.031	−0.277	0.032	0.299	0.021
Cys Arg Trp Trp	−0.276	0.033	−0.298	0.021	0.360	0.005
Phosphatidylethanolamine(32:3)	−0.250	0.054	−0.249	0.055	0.273	0.037
Sphingomyelin (D18: 0/14: 1 (9Z)(OH))	−0.247	0.057	−0.248	0.056	0.280	0.032
Trp Gln Asp Cys Glu	−0.256	0.049	−0.256	0.048	0.286	0.028
Tetrahydropteroyltri-L-glutamic acid	−0.264	0.042	−0.258	0.046	0.291	0.025
Phosphatidic acid PA(18:1(12Z)-2OH(9,10)/i-15:0)	−0.277	0.032	−0.283	0.028	0.357	0.006
Phosphatidylcholine(32:1)	−0.266	0040	−0.274	0.034	0.282	0.031
Phosphatidylethanolamine(34:2)	−0.259	0.045	−0.261	0.044	0.284	0.029
1-Methylinosine	−0.259	0.046	−0.260	0.045	0.285	0.029
Phosphatidylethanolamine(35:0)	−0.259	0.046	−0.260	0.045	0.278	0.033
Heptadecanoic carnitine	−0.257	0.047	−0.260	0.045	0.294	0.024
Phosphatidylcholine(36:0)	−0.264	0.042	−0.268	0.039	0.303	0.020
Phophatidylethanolamine(22:5)	−0.292	0.024	−0.295	0.022	0.296	0.023
Phosphatidylcholine(38:4)	−0.270	0.037	−0.276	0.033	0.301	0.021
Phosphatidic acid(40:1)	−0.259	0.046	−0.262	0.043	0.299	0.021
Phosphatidylcholine(34:1)	−0.253	0.051	−0.258	0.047	0.312	0.016
Phosphatidylserine(38:1)	−0.259	0.045	−0.265	0.041	0.301	0.021
Phophatidylethanolamine(44:9)	−0.272	0.035	−0.279	0.031	0.282	0.030
Phosphatidylcholine(22:2)	−0.258	0.047	−0.259	0.046	0.280	0.032
Phosphatidylcholine(42:6)	−0.250	0.054	−0.255	0.049	0.298	0.022
Phosphatidylcholine(40:3)	−0.255	0.049	−0.261	0.044	0.306	0.018
Phosphatidylethanolamine PGE2/22:2(13Z,16Z)	−0.258	0.046	−0.263	0.042	0.276	0.034
Phosphatidylcholine(42:2)	−0.276	0.033	−0.283	0.029	0.287	0.027
Phosphatidylcholine(44:6)	−0.257	0.047	−0.267	0.039	0.321	0.013

P¹: Spearman’s correlation coefficient; *p*-values were calculated from nonparametric Mann—Whitney test.

**Table 5:** Urinary metabolomic biomarkers related to oxidative stress and benzene-induced chromosomal aberrations.

Metabolite	*p* Value ^b^	Fold Change ^c^	Pathway
1,21-Henicosanediol	<0.0001	2.30	Lipid transport and lipid metabolism Fatty acid metabolism Lipid peroxidation and cell signaling.
Tetradecenoylcarnitine	<0.0001	5.05	Lipid transport and lipid metabolism Fatty acid metabolism Lipid peroxidation and cell signaling.
Coprocholic acid	<0.0001	5.33	Lipid transport and metabolism Fatty acid metabolism
Cys Met Thr Tyr	<0.0001	4.76	Product of incomplete decomposition of proteins or protein catabolism
Asp Leu	<0.0001	4.72	Product of incomplete decomposition of proteins or protein catabolism
Phenylalanylhydroxyproline	<0.0001	2.27	Product of incomplete decomposition of proteins or protein catabolism
Cys Hys Ser Trp	<0.0001	2.31	Product of incomplete decomposition of proteins or protein catabolism.
Sphingomyelin (D18: 0/14: 1 (9Z) (OH))	<0.0001	5.05	Lipid metabolism and signaling cell.
PE (PGE2/22:2 (13Z, 16Z))	<0.0001	4.73	Cell signaling
Phophatidylethanolamine(44:9)	<0.0001	5.20	Components of the lipid bilayer of cells Lipid metabolism Cell signaling

^b^: *p*-values were calculated from nonparametric Mann—Whitney test between occupational and environmental exposure groups. ^c^: fold change was calculated by the average concentration of the metabolite in the group with environmental exposure in relation to the group with occupational exposure.

## Data Availability

The datasets generated and analyzed in this current study are not publicly available for reasons of confidentiality. However, they are available from the corresponding author upon reasonable request.

## Supplementary Materials

The following supporting information can be downloaded at: https://www.mdpi.com/article/10.3390/metabo12100978/s1. Table S1: Descriptive analysis and comparison between groups for sociodemographic variables, Table S2: Time of exercise in the current occupation for workers in the environmental and occupational groups exposed to benzene, Table S3—Descriptive analysis and comparison between groups for biomarkers of exposure, effect, and biochemical and hematological parameters, Table S4— Correlation between candidate metabolites for exposure biomarkers and working time in the current occupation; Figure S1. Scatter diagrams between some metabolites and thiol and MDA, Figure S2. Scatter diagrams between some metabolites and chromosome breaks, fragment and metaphases presenting premature chromatid separation.
