# Supplementary material for: Metabolomic Study of Urine from Workers Exposed to Low Concentrations of Benzene by UHPLC-ESI-QToF-MS Reveals Potential Biomarkers Associated with Oxidative Stress and Genotoxicity
Source: Metabolites. 2022 Oct 16;12(10):978. doi: 10.3390/metabo12100978 (PMC9611274; doi:10.3390/metabo12100978)
Supplement: Supplementary file 1 [file metabolites-12-00978-s001.zip › metabolites-1981637-supplementary.pdf]

Supplementary materials

Table S1. Descriptive analysis and comparison between groups for sociodemographic variables.

Variables		General		Environmental		Occupational		p-value
		N=60	%	N=28	%	N=32	%	
Sex	Female	7	11.66%	4	14.29%	2	6.25%	0.2473
	Male	53	88.33%	24	85.71%	30	93.75%	
Age range	17-30 years	13	22.41%	3	11.53%	10	31.25%	0.0386
	30-40 years	14	24.13%	4	15.38%	10	31.25%	
	40-50 years	19	32.75%	13	50.00%	6	18.75%	
	>= 50 years	12	20.71%	6	23.09%	6	18.75%	
Tobacco use	Never smoked	37	61.70%	17	60.71%	20	62.50%	0.9796
	Used to smoke	15	25.00%	7	25.00%	8	25.00%	
	Smokers	8	13.30%	4	14.28%	4	12.50%	
Alcohol use	Never drank	14	23.33%	9	32.15%	5	15.63%	0.0789
	Used to drink	7	11.70%	5	17.85%	2	6.25%	
	Drinks	39	64.97%	14	50.00%	25	78.12%	

Note: 2 individuals from the environmental group did not inform their age.

Table S2. Time of exercise in the current occupation for workers in the environmental and occupational groups exposed to benzene.

Environmental group			
**Time (months)**	**Xi**	**Individuals number**	**Percentage (%)**
10 -77	43.4	6	27.27
78-145	111.2	3	13.64
146-212	179.0	6	27.27
213-280	246.8	5	22.73
281-348	314.6	2	9.09
**Total**		22	100
**Occupational group**			
**Time (months)**	**Xi**	**Individuals number**	**Percentage (%)**
12 -78,3	45.2	16	53.33
78,3 – 145	111.5	6	20.00
145- 211	177.8	1	3.33
211-277	244.2	3	10.00
277-343	310.5	1	3.33
343-210	376.8	3	10.00
**Total**		30	100

* Xi= average working time in current occupation in months

Table S3. Descriptive analysis and comparison between groups for biomarkers of exposure, effect and biochemical and hematological parameters.

**Variables**		**Group**		**p-value**
		**Environmental**	**Occupational**	
		**N = 28 Average (SD)**	**N= 32 Average (SD)**	
Exposure Biomarkers	SPMA (µg/gcreatinine)	1.25 (1.1)	1.79 (2.1)	0.2793
	A*tt*M (mg/gcreatinine)	0.19 (0.13)	0.22 (0.21)	0.6618
Complete blood count	Red Cells (10^6/mm^3)	4860312.50 (528805.60)	4723571.86 (429998.60)	0.4955
	Hb (g/dL)	14.00 (1.20)	13.40 (1.20)	0.0575
	HT (%)	40.40 (3.20)	42.40 (3.60)	**0.0253**
	MCV (fL)	86.50 (4.50)	87.80 (4.20)	0.2160
	MCH (pg)	29.70 (2.10)	30.0 (2.20)	0.6946
	MCHC (g/dL)	34.30 (1.10)	34.20 (1,70)	0.4109
	RDW (%)	12.0 (2.10)	11.70 (0.50)	**0.0120**
	Total leucocytes count (mm^3)	6341.8 (1553.6)	6391.8 (1915.9)	0.9127
	Neutrophils (%)	54.60 (7.50)	56.40 (10.10)	0.4339

	Neutrophils (mm^3)	3632.44 (1061.71)	3706.86 (1403.21)	0.6018
	Eosinophils (%)	4.30 (4.20)	3.10 (2.30)	0.4366
	Eosinophils (mm^3)	285,00 (320,50)	188.00 (137.80)	0.4633
	Basophils (%)	0.0 (0.0)	1.00 (0.90)	**0.0118**
	Basophils (mm^3)	0.0 (0.0)	55.90 (54.70)	**0.0000**
	Monocytes (%)	6.70 (2.40)	7.70 (1.60)	**0.0118**
	Monocytes (mm^3)	415.13 (126.99)	481.30 (141.10)	**0.0441**
	Lymphocytes (%)	34.40 (6.40)	31.80 (8.30)	0.1807
	Lymphocytes (mm^3)	2099.40 (515.60)	1983.30 (690.20)	0.4684
	Platelets (mm^3)	230214.30 (46124.90)	284184.40 (47964.7)	0.2565
Biochemical Markers	AST (U/L)	22.0 (9.00)	29.10 (11.90)	**0.0113**
	ALT (U/L)	27.30 (16.70)	22.40 (11.90)	0.2539
	Total Bilirubin (mg/dL)	0.37 (0.19)	0.46 (0,32)	0.3018
	Direct Bilirubin (mg/dL)	0.11 (0.06)	0.15 (0.08)	**0.0367**
	Indirect Bilirubin (mg/dL)	0.26 (0.13)	0.34 (0.23)	0.1307
	γ GT (U/L)	38.10 (24.72)	41.34 (30.0)	0.8764
	Rheumatoid Factor (UI/mL)	1.24 (0.66)	1.19 (0.49)	0.9941
Micronuclei	micronuclei (Mn/1000cells)	0.69 (0.53)	1.12 (1.19)	0.2329
Comet Assay	FPG-EC(%tail intensity)	39.46 (24.29)	35.03 (26.35)	0.5346
Oxidative Stress Markers	CAT (kU/L)	39.01 (13.63)	39.44 (28.23)	0.6132
	GST (kU/L)	13.57 (3.97)	17.09 (9.99)	0.9424
	Thiol (kU/L)	0.44 (0.05)	0.40 (0.09)	**0.0261**
	MDA (kU/L)	1.28 (0.76)	3.48 (4.39)	**0.0000**
	SOD (kU/L)	5.12 (18.08)	2.11 (1.21)	0.4104
Chromosomal aberrations	Number of chromosome breaks (breaks s/100cells)	1.16 (2.73)	8.06 (13.55)	**0.0017**
	Number of fragments (Frag/100 cells)	3.24 (5.46)	14.40 (25.94)	**0.0027**

Number metaphases with premature separation of chromatids (MCP/100cells)	2.48 (2.73)	2.12 (5.16)	**0.0303**

Hb=hemoglobin, HT=hematocrit, MCV=Mean Corpuscular Volume, MHC=Mean Corpuscular Hemoglobin, CHCM= Mean Corpuscular Hemoglobin Concentration, RDW= Distribution Range of Red Blood Cells,aspartate; Hb=hemoglobin, HT=hematocrit, MCV=Mean Corpuscular Volume, MHC=Mean Corpuscular Hemoglobin, CHCM= Mean Corpuscular Hemoglobin Concentration, RDW= Distribution Range of Red Blood Cells,= gamma glutamyl transferase; CAT= catalase; SOD = superoxide dismutase; THIOL= thiol groups; MDA= malondialdehyde (MDA); GST= glutathione S-transferase; S-PMA = *S*- phenylmercapturic acid; *tt*MA = *trans*,*trans*-muconic acid; C-FPG = enzyme formamidopyrimidine DNA glycosylase (C-FPG). The genotoxicity was measured by metaphases with chromosomal abnormalities (MCA) and nuclear abnormalities, comet assay using the enzyme formamidopyrimidine DNA glycosylase (C-FPG).

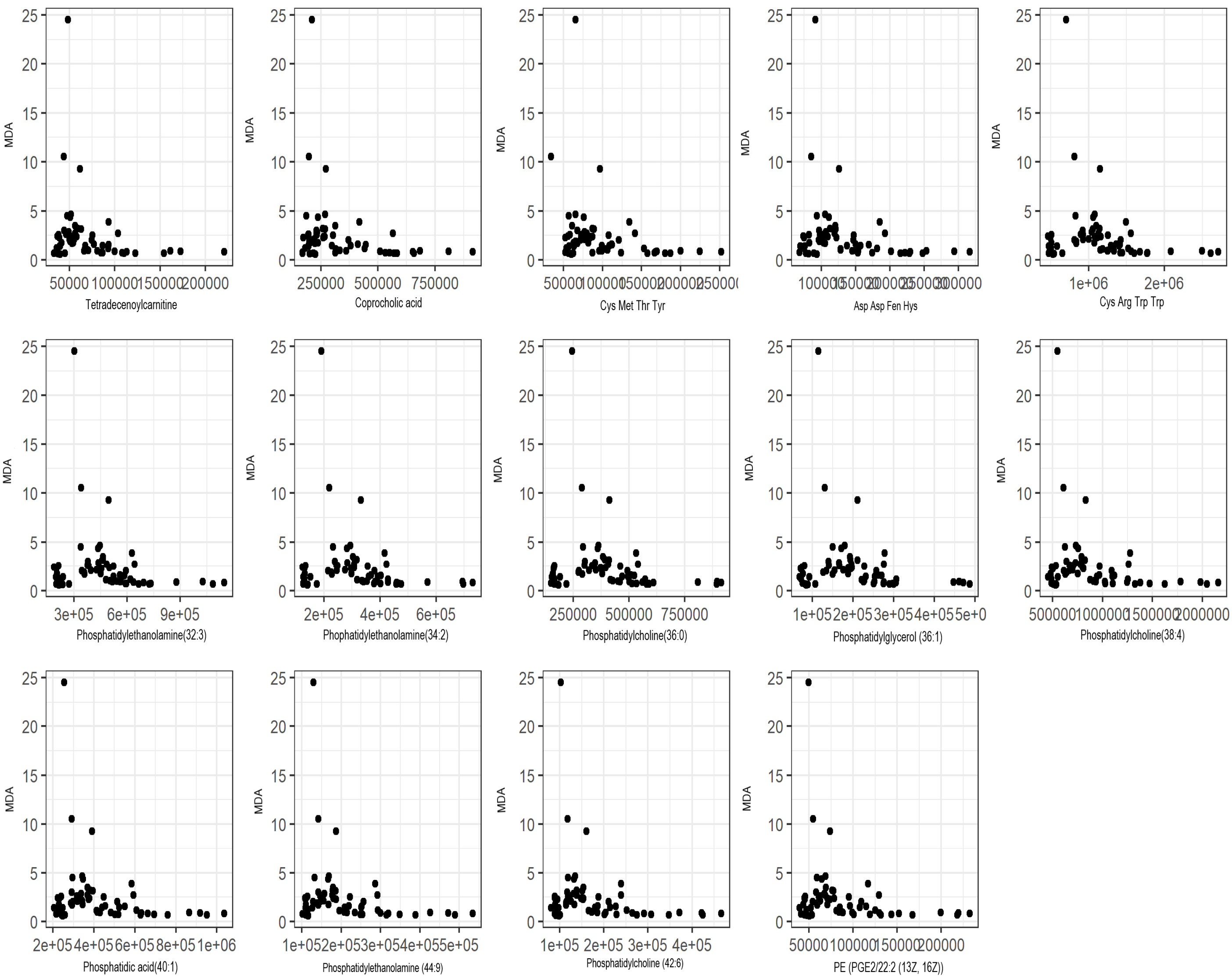

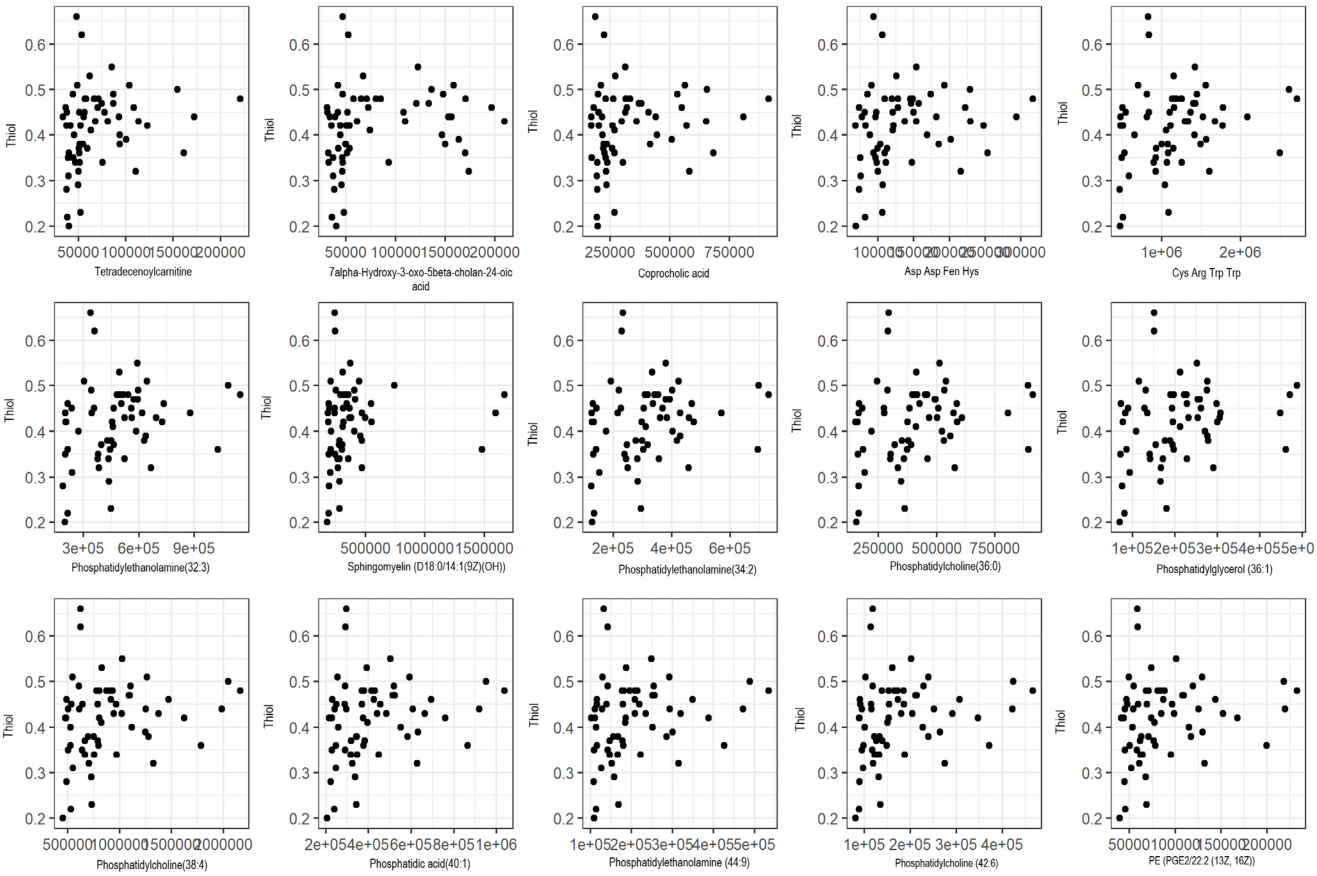

Figure S1. Scatter diagrams between some metabolites and Thiol and MDA.

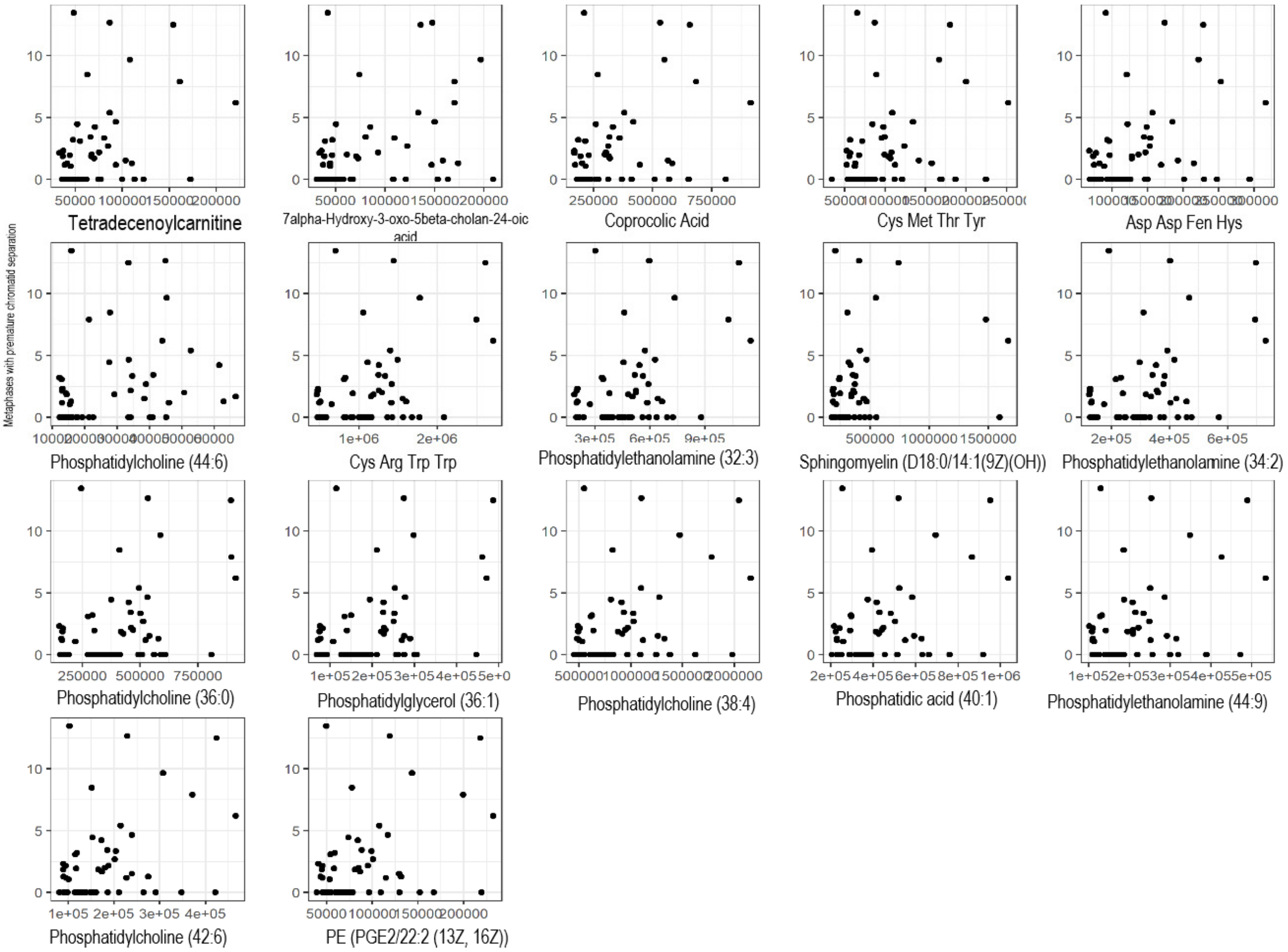
Metaphases with premature chromatid separation
Tetradecenoylcarnitine
7alpha-Hydroxy-3-oxo-5beta-cholan-24-oic acid
Coprocolic Acid
Cys Met Thr Tyr
Asp Asp Fen Hys
Phosphatidylcholine (44:6)
Cys Arg Trp Trp
Phosphatidylethanolamine (32:3)
Sphingomyelin (D18:0/14:1(9Z)(OH))
Phosphatidylethanolamine (34:2)
Phosphatidylcholine (36:0)
Phosphatidylglycerol (36:1)
Phosphatidylcholine (38:4)
Phosphatidic acid (40:1)
Phosphatidylethanolamine (44:9)
Phosphatidylcholine (42:6)
PE (PGE2/22:2 (13Z, 16Z))

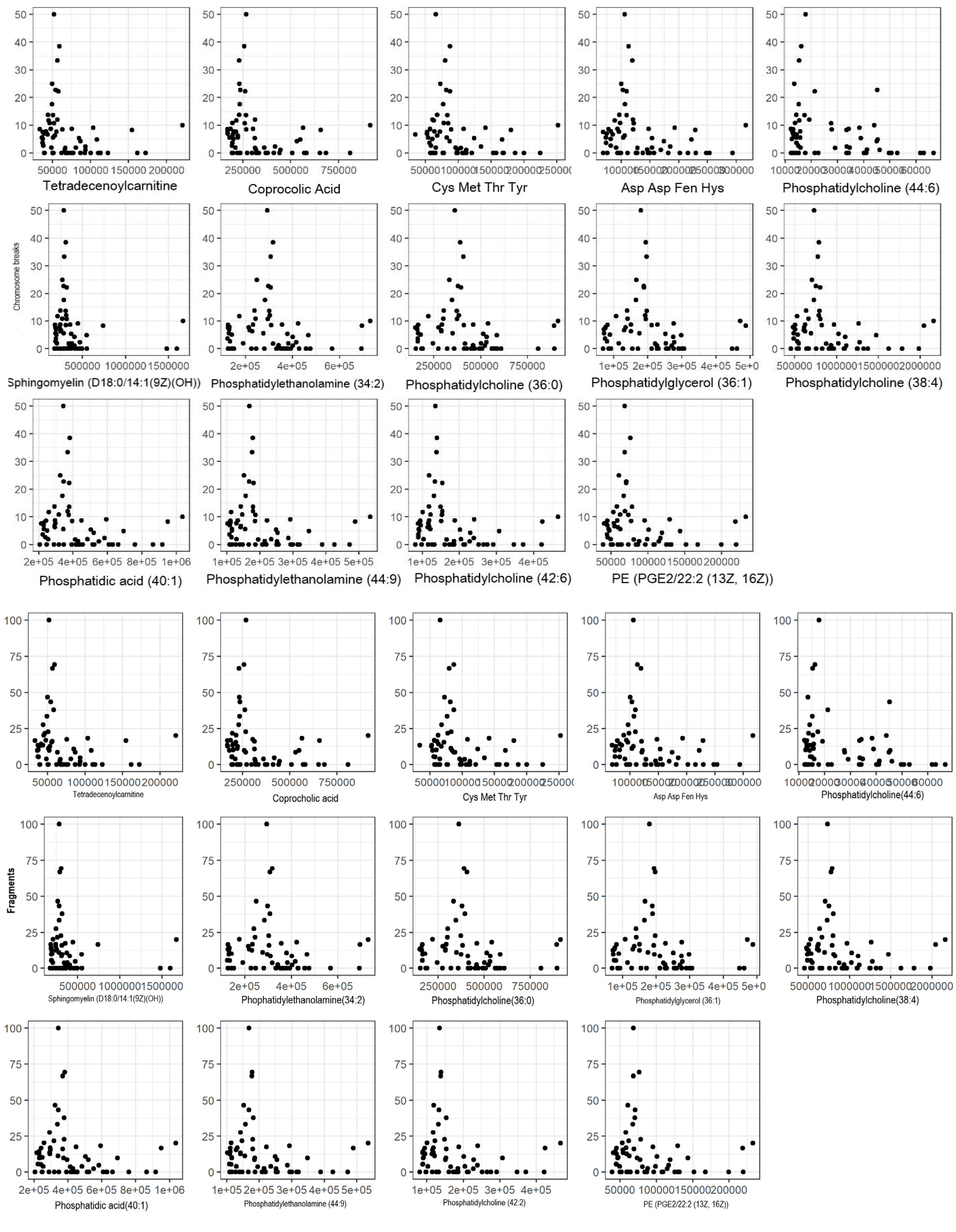

Figure S2. Scatter diagrams between some metabolites and chromosome breaks, fragment and metaphases presenting premature chromatid separation.

Table S4- Spearman correlation between urinary metabolites and oxidative stress markers.

	CAT		GST		THIOL		MDA		SOD	
	P[1]	p value	P[1]	p value	P[1]	p value	P[1]	p value	P[1]	p value
Phenylalanylhydroxyproline	-0.068	0.605	0.205	0.116	0.083	0.531	-0.342	0.007	-0.012	0.928
Sphingomyelin (d18:1/16:0)	-0.019	0.887	0.162	0.218	0.149	0.256	-0.356	0.005	-0.002	0.987
1,21-Henicosanediol	0.008	0.949	-0.017	0.897	0.047	0.721	0.007	0.957	0.007	0.960
Tetradecenoylcarnitine	0.114	0.386	0.264	0.041	0.314	0.014	-0.272	0.036	-0.215	0.099
7alpha-Hydroxy-3-oxo-5beta-cholan-24-oic acid	0.091	0.490	0.275	0.033	0.333	0.009	-0.216	0.098	-0.183	0.161
Testosterone glucuronide	0.034	0.796	0.268	0.039	0.273	0.035	-0.306	0.017	-0.267	0.039
Phosphatidylglycerol (36:1)	-0.146	0.267	-0.079	0.548	0.153	0.242	-0.083	0.528	0.274	0.034
Coprocholic acid	0.135	0.303	0.302	0.019	0.238	0.067	-0.305	0.018	-0.280	0.030
Folic acid	0.064	0.627	0.281	0.030	0.331	0.010	-0.276	0.033	-0.251	0.053
Asp Leu	0.097	0.459	0.246	0.058	0.236	0.069	-0.310	0.016	-0.223	0.087
Cys Met Thr Tyr	0.141	0.282	0.266	0.040	0.214	0.100	-0.303	0.019	-0.236	0.070
1-(9Z-heptadecenoyl)-2-(7Z, 10Z, 13Z, 16Z-docosatetraenoyl) -glycero-3-phosphoserine	0.086	0.514	0.295	0.022	0.281	0.030	-0.275	0.034	-0.260	0.045
Cys Hys Ser Trp	0.030	0.817	0.275	0.033	0.254	0.051	-0.248	0.056	-0.261	0.044
Asp Asp Fen Hys	0.054	0.680	0.287	0.026	0.283	0.028	-0.246	0.059	-0.275	0.034
Cys Arg Trp Trp	0.044	0.739	0.292	0.024	0.247	0.057	-0.196	0.132	-0.401	0.002
Phosphatidylethanolamine(32:3)	0.113	0.391	0.297	0.021	0.291	0.024	-0.265	0.041	-0.262	0.043
Sphingomyelin (D18: 0/14: 1 (9Z) (OH))	0.103	0.433	0.308	0.017	0.303	0.019	-0.265	0.041	-0.264	0.042

Trp Gln Asp Cys Glu	0.087	0.511	0.312	0.015	0.284	0.028	-0.256	0.049	-0.270	0.037
Tetrahydropteroyltri-L-glutamic acid	0.039	0.770	0.294	0.022	0.321	0.012	-0.330	0.010	-0.213	0.103
Phosphatidic acid PA(18:1(12Z)-2OH(9,10)/i-15:0)	-0.005	0.969	0.300	0.020	0.240	0.065	-0.348	0.006	-0.278	0.031
Phosphatidylcholine(32:1)	0.095	0.472	0.304	0.018	0.268	0.038	-0.253	0.051	-0.256	0.048
Phosphatidylethanolamine(34:2)	0.096	0.464	0.306	0.017	0.286	0.027	-0.258	0.047	-0.259	0.045
1-Methylinosine	0.107	0.414	0.295	0.022	0.277	0.032	-0.256	0.049	-0.261	0.044
Phosphatidylethanolamine(35:0)	0.121	0.358	0.291	0.024	0.274	0.034	-0.253	0.051	-0.251	0.053
Heptadecanoic carnitine	0.081	0.539	0.293	0.023	0.291	0.024	-0.269	0.038	-0.262	0.043
Phosphatidylcholine(36:0)	0.064	0.625	0.301	0.019	0.290	0.025	-0.266	0.040	-0.252	0.052
Phophatidylethanolamine(22:5)	0.096	0.468	0.297	0.021	0.268	0.038	-0.255	0.049	-0.279	0.031
Phosphatidylcholine(38:4)	0.091	0.489	0.308	0.017	0.266	0.040	-0.256	0.048	-0.251	0.053
Phosphatidic acid(40:1)	0.090	0.493	0.299	0.021	0.287	0.026	-0.273	0.035	-0.253	0.051
Phosphatidylcholine(34:1)	0.093	0.480	0.295	0.022	0.275	0.034	-0.261	0.044	-0.262	0.043
Phosphatidylserine(38:1)	0.088	0.503	0.296	0.022	0.277	0.032	-0.259	0.045	-0.252	0.052
Phophatidylethanolamine(44:9)	0.099	0.454	0.307	0.017	0.285	0.028	-0.257	0.047	-0.257	0.047
Phosphatidylcholine(22:2)	0.089	0.501	0.304	0.018	0.294	0.023	-0.262	0.043	-0.262	0.043
Phosphatidylcholine(42:6)	0.100	0.448	0.299	0.020	0.289	0.025	-0.256	0.048	-0.242	0.063
Phosphatidylcholine(40:3)	0.082	0.536	0.288	0.026	0.288	0.026	-0.261	0.044	-0.257	0.048
Phosphatidylethanolamine PGE2/22:2(13Z,16Z)	0.095	0.473	0.289	0.025	0.263	0.043	-0.281	0.029	-0.238	0.067
Phosphatidylcholine(42:2)	0.079	0.547	0.311	0.015	0.285	0.027	-0.274	0.034	-0.279	0.031
Phosphatidylcholine(44:6)	0.075	0.569	0.310	0.016	0.293	0.023	-0.270	0.037	-0.252	0.052

P[1]: Spearman's Correlation Coefficient; p-values were calculated from nonparametric Mann–Whitney test. Note: S-phenilmercapturic Acid (SPMA) and *trans-trans*-muconic acid (*t,t*-MA).

Table S5. Correlation between candidate metabolites for exposure biomarkers and working time in the current occupation.

Metabolites	p-value	P[1]
1,21-Henicosanediol	0.185	-0.106
Tetradecenoylcarnitine	0.765	-0.040
Coprocholic acid	0.880	0.020
Cys Met Thr Tyr	0.774	-0.037
Asp Leu	0.544	-0.079
Phenylalanylhydroxyproline	0.428	-0.105
Cys Hys Ser Trp	0.396	-0.111
Sphingomyelin (D18: 0/14: 1 (9Z) (OH))	0.5071	-0.087
PE (PGE2/22:2 (13Z, 16Z))	0.637	-0.010
Phophatidylethanolamine(44:9)	0.567	-0.075

P[1] = Spearman's Correlation Coefficient.
